# Co-activation of Sonic hedgehog and Wnt signaling in murine retinal precursor cells drives ocular lesions with features of intraocular medulloepithelioma

**DOI:** 10.1038/s41389-021-00369-0

**Published:** 2021-11-16

**Authors:** Matthias Dottermusch, Piotr Sumisławski, Julia Krevet, Maximilian Middelkamp, Hannah Voß, Bente Siebels, Harald Bartsch, Karl Sotlar, Peter Meyer, Stephan Frank, Andrey Korshunov, Markus Glatzel, Ulrich Schüller, Julia E. Neumann

**Affiliations:** 1 Center for Molecular Neurobiology (ZMNH), University Medical Center Hamburg-Eppendorf, Hamburg, Germany; 2 Institute of Neuropathology, University Medical Center Hamburg-Eppendorf, Hamburg, Germany; 3 Center for Neuropathology, Ludwig Maximilians University, Munich, Germany; 4 Research Institute Children’s Cancer Center Hamburg, Hamburg, Germany; 5 Institute of Clinical Chemistry and Laboratory Medicine, Mass Spectrometric Proteomics, University Medical Center Hamburg-Eppendorf, Hamburg, Germany; 6 Department of Pathology, Ludwig Maximilians University, Munich, Germany; 7 Department of Pathology, University Hospital, Paracelsus Medical University, Salzburg, Austria; 8 Department of Ophthalmology, University Hospital Basel, University of Basel, Basel, Switzerland; 9 Division of Neuropathology, Institute of Medical Genetics and Pathology, University Hospital Basel, University of Basel, Basel, Switzerland; 10 Clinical Cooperation Unit Neuropathology (G380), German Cancer Research Center (DKFZ), Heidelberg, Germany; 11 Department of Pediatric Hematology and Oncology, University Medical Center Hamburg-Eppendorf, Hamburg, Germany

**Keywords:** Cancer models, Embryonal neoplasms, CNS cancer

## Abstract

Intraocular medulloepithelioma (IO-MEPL) is a rare embryonal ocular neoplasm, prevalently occurring in children. IO-MEPLs share histomorphological features with CNS embryonal tumors with multilayered rosettes (ETMRs), referred to as intracranial medulloepitheliomas. While Sonic hedgehog (SHH) and WNT signaling pathways are crucial for ETMR pathogenesis, the impact of these pathways on human IO-MEPL development is unclear. Gene expression analyses of human embryonal tumor samples revealed similar gene expression patterns and significant overrepresentation of SHH and WNT target genes in both IO-MEPL and ETMR. In order to unravel the function of Shh and Wnt signaling for IO-MEPL pathogenesis in vivo, both pathways were activated in retinal precursor cells in a time point specific manner. Shh and Wnt co-activation in early *Sox2-* or *Rax*-expressing precursor cells resulted in infiltrative ocular lesions that displayed extraretinal expansion. Histomorphological, immunohistochemical, and molecular features showed a strong concordance with human IO-MEPL. We demonstrate a relevant role of WNT and SHH signaling in IO-MEPL and report the first mouse model to generate tumor-like lesions with features of IO-MEPL. The presented data may be fundamental for comprehending IO-MEPL initiation and developing targeted therapeutic approaches.

## Introduction

Intraocular medulloepithelioma (IO-MEPL) is an embryonal ocular neoplasm, which mainly affects children. IO-MEPLs commonly grow in the ciliary body but are also found in the retina or optic nerve [1]. In advanced growth stages, IO-MEPL is standardly treated by primary enucleation, which generally results in a good overall prognosis for most patients [2]. As of today, targeted therapeutic approaches with the aim of subsequent eye salvage remain to be explored.

Based on occurrence and histomorphology, IO-MEPLs are believed to arise from primitive medullary epithelium of the optic cup [3, 4]. Characteristic histomorphology strongly resembles neuroepithelium of the embryonic neural tube and occasionally displays pigmented epithelial cells as well as teratoid and stromal elements [5]. Many of these features also reflect the typical morphology found in variants of embryonal tumors with multilayered rosettes (ETMR) [2, 6]. These variants of ETMRs are referred to as intracranial medulloepitheliomas, and, in contrast to IO-MEPL, represent highly aggressively growing pediatric brain tumors with specific molecular characteristics [5, 7].

We have recently described enrichment of Sonic hedgehog (SHH) and WNT signaling pathways as a characteristic molecular feature of ETMRs [8]. Moreover, Shh and Wnt activation is sufficient to induce cerebral tumors with compatible ETMR characteristics in mice [8]. Of note, in single cases of IO-MEPLs, mutations in *PTCH1*, a component of the SHH pathway have also been described [9]. With respect to the resemblance of ETMR and IO-MEPL, our objective in this study was to investigate human IO-MEPL for SHH and WNT pathway activation and to test the effect of synchronous Shh and Wnt activation in a murine model system for early eye development.

## Methods

### Human tissue

Twenty-five formalin-fixed and paraffin-embedded (FFPE) human tumor tissue biopsy samples comprising six different tumor entities (8 IO-MEPL; 3 ETMR; 5 SHH-MB; 4 WNT-MB; 4 Group 4-MB; a tissue microarray (TMA, *n* = 182) of retinoblastoma) were available and analyzed in this study. ETMR and medulloblastomas (MBs) were obtained from the Center for Neuropathology, Ludwig Maximilians University (LMU), Munich. The retinoblastoma TMA was obtained from the Division of Neuropathology, Basel University Hospital. IO-MEPLs have been published before [9]. All patients gave their informed consent for scientific use of the data. Approval was obtained from the institutional review boards of the respective medical associations.

### Animals

*hGFAP-cre* [10], *Sox2-creER*^*T2*^ [11], *SmoM2* ^*fl/fl*^ [12], *Rax-creER*^*T2*^ [13], and *Gt(ROSA)26Sor*^*tm1Hjf*^ (*R26-lsl-RFP*) [14] mice were purchased from The Jackson Laboratories (Bar Harbor, ME, USA). *Ctnnb1(ex3)*^*fl/fl*^ mice [15] were generously provided by Mark Taketo (University of Tokyo, Tokyo, Japan). In these mice, Cre-induced recombination leads to removal of exon 3, which results in a stabilized beta-Catenin protein, which mimics constitutive Wnt signaling [16]. For co-activation experiments, a *Ctnnb1(ex3)*^*fl/fl*^*SmoM2*^*fl/fl*^ strain was generated. Both male and female mice were examined.

In timed mating experiments, the day of vaginal plug detection was considered E0.5. Pregnant females were injected with tamoxifen (solubilized in corn oil, T5648, Sigma-Aldrich) at E8.5, 10.5, 12.5, or 14.5 via intraperitoneal injection. Tamoxifen was administered either once with 1 mg or twice with 0.6 mg per injection and a 24-h time interval. Pregnant females were monitored until E18.5, when all embryos were collected for examination. Local governmental animal care regulations limited analyses to prenatal time points only. All experiments using animals were approved by the local animal care committee (Behörde für Justiz und Verbraucherschutz in Hamburg) and handling was conducted in accordance with local governmental and institutional animal care regulations. No randomization or blinding was performed in this study. All generated biological replicates were analyzed and incorporated in this study.

### Genotyping

DNA was extracted from ear or tail biopsies using Laird’s buffer (10 mM Tris-HCl pH 8.5, 5 mM EDTA, 0.5% SDS, 0.2 M NaCl, 0,1 mg/ml protein kinase K in ddH_2_O) and isopropanol precipitation. DNA was dissolved in DEPC-treated water and stored at 4 °C. Genotype-specific regions of the genome were amplified via PCR utilizing the following primers (*Cre*: TCCGGGCTGCCACGACCAA, GGCGCGGCAACACCATTTT, *Smo*: CTTGGGTGGAGAGGCTATTC, AGGTGAGATGACAGGAGATC, *Ctnnb1*: CGTGGACAATGGCTACTCAA, TGTCCAACTCCATCAGGTCA, *RFP*: AAAGTCGCTCTGAGTTGTTAT, GCGAAGAGTTTGTCCTCAACC, GGAGCGGGAGAAATGGATATG) and a DreamTaq-Polymerase (EP0703, Thermo Fisher Scientific, Waltham, MA, USA) based standard reaction mixture.

### Microscopy and immunohistochemistry

Tissue samples were fixed in 4% paraformaldehyde, dehydrated, embedded in paraffin, and then sectioned at 2 µm according to standard laboratory protocols. Immunohistochemical staining was performed on an automated staining machine (Ventana BenchMark TX, Roche Diagnostics, Mannheim, Germany). The following primary antibodies were used: Calretinin (610908, BD Biosciences, 1:1000), Caspase 3 (AF835, R&D Systems, 1:300), GFAP (M0761, Dako 1:200), Ki67 (ab15580, Abcam, 1:100), LIN28A (3978, Cell Signaling Technology, 1:100), MAP2C (M4403, Sigma-Aldrich, 1:3000), Nestin (611658, BD Biosciences, 1:200), OLIG2 (A9610, Millipore, 1:200), OTX2 (MA5-15854, Invitrogen, 1:2000), pHH3 (9706L, Cell Signaling Tech, 1:200), SOX2 (ab79351, Abcam, 1:200), Vimentin (ab92547, Abcam, 1:200), RFP (ABIN129578, Antibodies online, 1:50). Detection was performed with secondary antibodies and diaminobenzidine (DAB) as a chromogen.

### RNA extraction

RNA from FFPE human tumor tissue biopsy samples (8 IO-MEPL, 3 ETMR, 5 SHH-MB, 4 WNT-MB, 4 Group 4-MB) was extracted using the High Pure RNA Isolation Kit (06650775001, Roche Diagnostics). RNA was extracted from fresh frozen murine tissue samples (3 cerebellar tumors of 14-day-old *hGFAP-cre::SmoM2*^*fl/+*^ *mice*, 5 eyes of *Rax-creER*^*T2*^*::Ctnnb1(ex3)*^*fl/+*^*SmoM2*^*fl/+*^ (*RBS*) and 4 eyes of *Ctnnb1(ex3)*^*fl/+*^*SmoM2*^*fl/+*^ (*BS*) mice as controls) using the RNeasy® Mini Kit (74136, Qiagen, Hilden, Germany). One eye per mouse was used for RNA extraction. Phenotypes were confirmed by hematoxylin–eosin histology of the contralateral eye.

### Nanostring analysis

A minimum of 100 ng RNA per human tissue sample was analyzed using the nCounter PanCancer pathways panel on the nCounter FLEX Analysis System (NanoString Technologies, Seattle, Washington, USA) at the Institute of Pathology of the LMU in Munich.

A minimum of 30 ng RNA per mouse tissue sample was analyzed using the nCounter Mouse PanCancer pathways panel on the nCounter SPRINT Profiler System (NanoString Technologies) at the Nanostring Core Facility of the University Medical Center Hamburg-Eppendorf in Hamburg.

Nanostring gene expression data was compiled, normalized, and log2-transformed using the nSolver Analysis Software 4.0.70 including the nCounter Advanced Analysis Software 2.0.115.

### Extraction and processing of proteins

FFPE tissue of murine embryonal tumors of E18.5 *GFAP-cre::Ctnnb1(ex3)*^*fl/+*^*SmoM2*^*fl/+*^ (*GBS*, *n* = 2) mice, as well as eyes of *RBS* embryos subjected to tamoxifen on E8.5 (*n* = 2), E10.5 (*n* = 2), E12.5 (*n* = 2), and E14.5 (*n* = 2) and eyes of *BS* littermates (*n* = 8) were deparaffinized using n-heptane and 70% ethanol and treated with 0.1 M TEAB with 1% SDC for 1 h at 99 °C prior to sonification. Protein concentrations were determined using the Pierce BCA Protein assay kit (Thermo Scientific). For tryptic digestion, 10 μg protein of each sample were used as previously described [17].

### Liquid-chromatography-coupled tandem mass spectrometer (LC-MS/MS)-based proteomic analysis

Prior to mass spectrometric analyses, peptides were resuspended in 0.1% formic acid. In all, 1 µg was used for measurement of every sample. LC-MS/MS measurements were performed on a Q Exactive Hybrid Quadrupole-Orbitrap Mass Spectrometer (Thermo Scientific) with nano-UPLC (Dionex Ultimate 3000, Thermo Scientific). Raw spectra were searched against the FASTA database (August 2021) using SEQUEST algorithm, implemented in the Proteome Discoverer Software v2.4.1.15.

Protein data were log2-transformed and samples were normalized via median centering. For every protein, median values of corresponding experimental and control groups were calculated.

### Unsupervised hierarchical clustering

Gene expression and protein data was analyzed using MultiExperiment Viewer (MeV) v4.9.0 software [18] (https://www.tm4.org). Distance method used was Pearson correlation, dendrogram drawing method used was average linkage. To visualize expression data in *z*-score format, each row was mean centered and divided by the standard deviation.

### Principle component analysis (PCA)

PCA was performed using Perseus v1.6.15 [19]. PCA plots were generated using GraphPad Prism v8.4 (GraphPad Software, San Diego, CA, USA).

### Gene set enrichment analysis (GSEA)

GSEA was performed on normalized count gene expression data using the GSEA software v4.0.3 of the Broad Institute [20, 21]. Gene sets were compiled as predefined by NanoString Technologies’ nCounter® (Mouse) PanCancer pathways panel (https://www.nanostring.com/support-documents/ncounter-pancancer-human-pathways-panel-gene-list/?dl=1). Gene sets with a size <15 or >100 genes were excluded from the analysis. Genes were ranked by log2 ratio of classes or diff of classes (for log-scale data) and statistical significance was determined by 1000 gene set permutations. *p* < 0.05 was considered significant.

### Data harmonization

For merging of internal and external datasets as well as cross-species analysis, human and murine datasets were reduced to homologous genes. Batch effects were removed using the parametric empirical Bayes framework provided by the ComBat algorithm [22] of the sva package [23] in R-3.6.3 [24].

### Agreement of differential expression (AGDEX)

In order to compare the transcriptome of human and murine tumor samples, the AGDEX statistic implemented in C++ was applied as previously published [25, 26]. *p* < 0.05 was considered significant.

### Statistical analysis and figures

Differentially expressed genes between two sample groups were determined by *t* test (Welch correction, 100 permutations, critical 0.05, adjusted Bonferroni) on log2-transformed gene expression data in MeV v4.9.0.

Differentially expressed genes between more than two sample groups were determined by analysis of variance test (1000 permutations, critical 0.01, adjusted Bonferroni) on log2-transformed gene expression data in MeV v4.9.0.

The sample-specific coefficient of variation, sample-specific mean, and inter-sample Pearson correlation coefficients were computed and visualized in GraphPad Prism v8.4.3. Venn diagrams were generated using DeepVenn v1 [27]. Graphs and images were formatted into figures using Adobe Illustrator 25.2.1.

### Nuclear staining quantification

Immunostained slides were digitalized using a Hamamatsu NanoZoomer 2.0-HT C9600 whole slide scanner. Whole images of control eyes and *RBS* lesions were exported using the NDP.view v2.7.43 software with consistent resolution (×40 lens) settings. Exported images were white balanced in Adobe Photoshop Version 22.3.1 prior to quantification.

The color deconvolution plugin in Fiji/ImageJ [28] was used to separate images into hematoxylin (H), DAB, and residual color channels.

For quantification and segmentation of nuclear structures, global thresholding was performed on the different color channels. In detail, the global threshold was gradually lowered in a looping statement, while the thresholded image was simultaneously analyzed and screened for particles holding features compatible with nuclei. The criteria for these features were dynamically adjusted by the iteration of the loop and comprised a pixel area of at least 27 to a maximum of 55 pixels, as well as a circularity ranging from 0.07 to 1.00. When compatible particles were found, these were counted and replaced by empty pixels.

For segmentation of positive nuclei, this approach was performed using the DAB-channel. For segmentation of negative nuclei, a combinatory image was used, which was obtained by adding the inverted DAB-channel to the sum of the H and the residual channel in the Image Calculator plugin. For segmentation of any remaining negative nuclei, a grayscale converted version of the original H/DAB staining image was used.

Tissue areas not eligible for quantification were excluded from the analysis. To review the validity of the digitally supported analysis approach, overlay images with the identified particles colored in red and blue (positive and negative nuclei, respectively) were created. All generated overlay images were determined as adequate.

### Gene ontology (GO) term network analysis

GO term networks were generated in Cytoscape 3.8.2 [29] and its integrated applications ClueGo 2.5.7 and CluePedia 1.5.7 [30, 31] using the REACTOME Reactions #12559 (11188) database. GO Term/Pathway Selection was defined as minimum 2 genes and 2% genes for human data and 8 genes and 20% genes for murine data. GO Term/Pathway Network Connectivity (Kappa score) was set to a minimum of 0.3. Statistical test used was enrichment/depletion (two-sided hypergeometric test). Multiple testing correction method used was Bonferroni step down. Pathways with *p* > 0.05 were excluded from the network. GO group affiliations of terms were represented by node colors.

Representative words for node clusters, were determined using the application Wordcloud [32] with normalization to the entire network with weight 0.2.

### RNA meta-analysis

CEL files from the two datasets GSE30074 [33] and GSE172170 [34] of the same array (Affymetrix Human Gene 1.0 ST) were jointly processed and normalized using the TAC software v4.0.2.15 (Thermo Scientific). MB molecular subgroups were assigned to the cases of GSE30074 according to GSE124814 [35].

The merged dataset was collapsed using Broad institute software and reduced to 703 common homologous genes shared with the human and mouse Nanostring PanCancer pathways panels. In order to compile datasets of similar sizes, 11 MB and 8 retinoblastoma samples were randomly selected for further analysis. First, internal and external datasets of human tumors were harmonized for batch effects using the ComBat algorithm. Subsequently, human and murine datasets were likewise combined.

### Data accession

Nanostring RNA expression data of human and mouse samples are accessible via GEO accession numbers GSE173758 and GSE173763.

Mouse protein data as well as experimental and processing details are accessible via the accession number PXD028697 of the PRIDE database [36] (ProteomeXchange Consortium).

## Results

### SHH and WNT signaling are co-activated in IO-MEPL

**Fig. 1:**
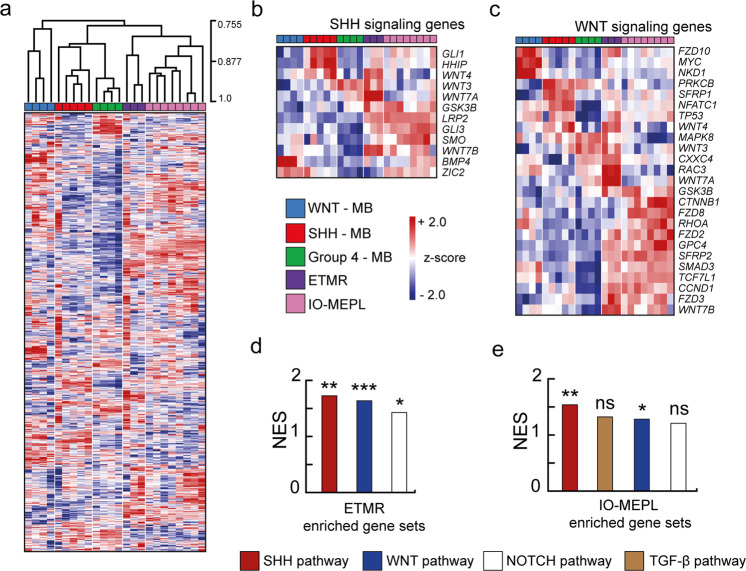
Gene expression analysis of IO-MEPLs and intracranial embryonal tumors. **a** Unsupervised hierarchical clustering based on expression of 770 genes of the PanCancer pathways panel (Nanostring Technologies). ETMRs and IO-MEPLs formed individual groups within a distinct shared dendrogram branch that separates the entities from molecular medulloblastoma subgroups. Distance method used was Pearson correlation; dendrogram drawing method used was average linkage. **b**, **c** Expression profiles based on differentially expressed genes of the Sonic hedgehog (SHH, **b**) and WNT (**c**) pathways displayed common upregulated target genes in IO-MEPLs and ETMRs (ANOVA test with adjusted Bonferroni correction). SHH and WNT gene sets were defined by the PanCancer pathways panel. Note the robust upregulation of *CTNNB1* and *SMO* in IO-MEPLs. **d**, **e** Gene set enrichment analysis (GSEA) reveals overrepresentation of both Wnt and Shh signaling pathway genes in ETMRs (**d**) as well as IO-MEPLs (**e**). Analysis was performed based on predefined Nanostring gene sets with Group 4-MBs used as a reference. Gene sets with an FDR value <0.25 are shown. Asterisks indicate significance: *p* value < 0.05*, <0.01**, <0.001***.

In order to unravel pathway alterations in IO-MEPLs, we analyzed gene expression data from tumor samples of 8 previously genetically characterized human IO-MEPLs [9] along with a set of 16 intracranial embryonal tumors comprising ETMRs and MBs, including the molecular groups SHH-, WNT-, and Group 4-MB. Unsupervised hierarchical clustering demonstrated primary clustering of the samples within their distinct tumor entity groups. Furthermore, IO-MEPL and ETMR tumors shared a common dendrogram branch (Fig. 1a), thus demonstrating similarities in gene expression. In order to search for tumor-specific cancer signaling pathways we constructed gene sets based on the predefined cancer-associated canonical pathways of the Nanostring PanCancer pathways panel. Significantly differentially expressed genes of SHH and WNT gene sets between tumor entities are illustrated in Fig. 1b, c. Of note, the highest mean expression values for both *SMO* and *CTNNB1* were found in the group of IO-MEPLs. Enrichment of SHH and WNT signaling genes in ETMR and IO-MEPL was analyzed using GSEA. Of the gene sets included in the analysis, the pathways SHH (*p* = 0.001), WNT (*p* < 0.001), and NOTCH (*p* = 0.035) were identified as significantly enriched in ETMRs (Fig. 1d). When correspondingly analyzing IO-MEPLs, SHH (*p* = 0.004) and WNT (*p* = 0.046) pathways were likewise and exclusively identified as significantly enriched (Fig. 1e).

### Comparative gene expression analysis of ETMRs and IO-MEPLs

**Fig. 2:**
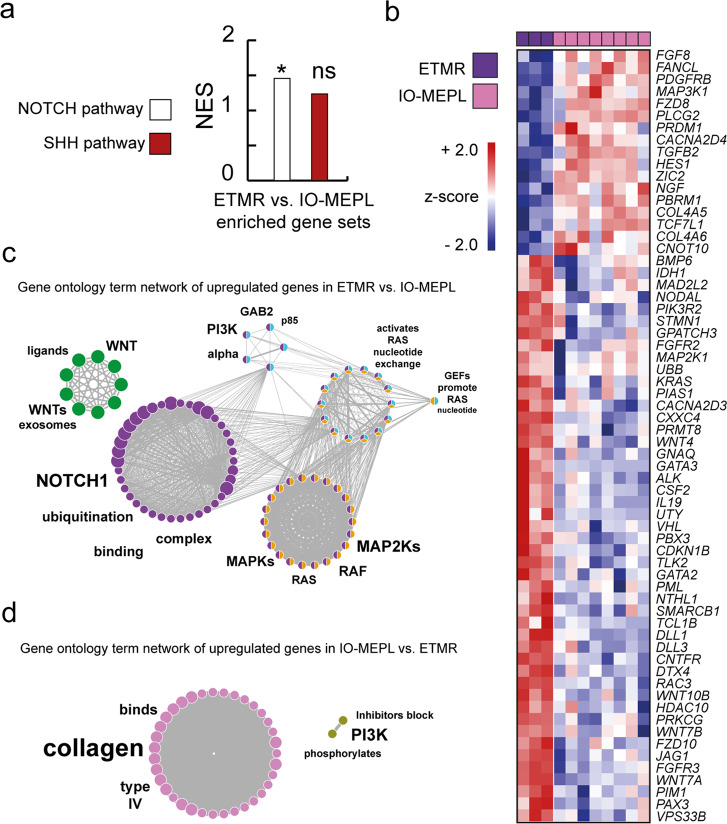
Differential gene expression and functional network analysis of IO-MEPLs compared to ETMRs. **a** Notch pathway genes were overrepresented in ETMR in comparison to IO-MEPL (GSEA). Gene sets with an FDR value <0.25 are shown. Asterisk indicates significance: *p* value < 0.05*. **b** Differentially expressed genes in IO-MEPLs compared to ETMRs. Welch-corrected *t* test with adjusted Bonferroni correction. **c**, **d** Gene ontology (GO) term network of upregulated genes in ETMR compared to IO-MEPL (**c**) and vice versa (**d**). Each node represents a significant GO term. Node colors indicate affiliation of GO terms to GO groups. Node clusters are annotated with the top four most significant words of the respective GO term aggregation. Font size differences within the annotation of a distinct cluster represent varying significances. Edges indicate term-term interrelations with Kappa score >0.3.

In order to explore differences in molecular characteristics of ETMRs and IO-MEPLs, we performed GSEA between the two tumor groups (Fig. 2a). GSEA demonstrated the NOTCH pathway gene set to be significantly enriched in ETMRs compared to IO-MEPLs (*p* = 0.036). Furthermore, we identified significantly differentially expressed genes in ETMRs and IO-MEPLs (Fig. 2b). GO term networks were generated based on the upregulated genes in ETMRs and IO-MEPLs and vice versa (Fig. 2c, d and Supplementary Tables 1 and 2). Among the terms illustrated, NOTCH signaling was again confirmed as a molecular characteristic of ETMRs, while associated terms with type IV collagens were linked to IO-MEPLs.

In summary, ETMRs and IO-MEPLs showed a co-activation of SHH and WNT signaling pathways, but gene expression revealed differences in NOTCH signaling pathway activation and functions associated with type IV collagens.

### Co-activation of Wnt and Shh signaling in vivo

Next, we asked whether Shh and Wnt signaling are crucial for the development of IO-MEPL. In order to test this hypothesis, we turned to an in vivo system and analyzed the effect of Shh and Wnt activation in retinal precursor cells during distinct time points of embryonal ocular development.

**Fig. 3:**
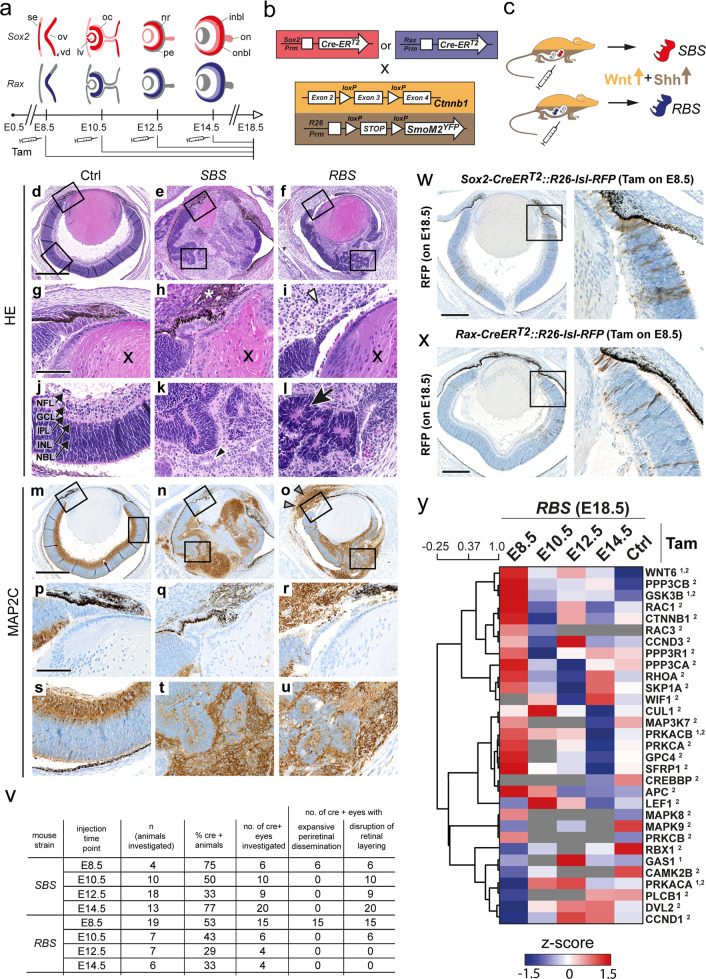
Time point-specific Shh and Wnt co-activation in *Sox2*- or *Rax*-positive retinal progenitor cells during embryonic development. **a** Scheme of *Sox2* and *Rax* expression during eye development based on previously published data [37, 38, 52–57]. At early developmental stages, the head ectoderm initially expresses low levels of *Sox2*. Its contact with the optic vesicle elicits local augmentation of *Sox2* expression [56]. Around E9.5, *Rax* expression is observed in the ventral diencephalon as well as the optic evaginations [52]. At E10.5, the closing lens vesicle and the inner layer of the optic cup express *Sox2* strongly, while the surface ectoderm and the outer layer of optic cup are low in *Sox2* expression [56]. In later stages, *Sox2* expression in the lens decreases and becomes confined to the lens epithelium, while strong *Sox2* expression is retained in the retina during further developmental stages and is also found in the optic nerve [53, 54]. In comparison, *Rax* expression is more strongly restricted to the developing retina at earlier stages and also develops a decreasing gradient from the inner to the outer retinal layers [55]. *Rax* expression has also been reported in extraocular tissue, namely, the hypothalamus, pituitary gland, and pineal gland during embryonic stages in rodents [38]. In contrast, *Sox2* is generally more extensively expressed and remains detectable throughout various tissues [57]. Tamoxifen administration was initiated at E8.5, E10.5, E12.5, or E14.5. Ocular development continued until E18.5, when embryos were investigated. Colors with higher opacity indicate relatively lower or diminishing expression. Gray color indicates very low or insignificant expression. se surface ectoderm, ov optic vesicle, vd ventral diencephalon, lv lens vesicle, oc optic cup, nr neural retina, pe pigmented epithelium, inbl inner neuroblast layer, onbl outer neuroblast layer, on optic nerve. **b** Breeding scheme used in this study: Mice expressing CreER^T2^ either under the *Sox2* or the *Rax* promoter were mated with mice carrying both a *Ctnnb1* gene with exon 3 flanked by *loxP* sites and a *SmoM2* construct following a *loxP* flanked functional “STOP” sequence containing a polyadenylation sequence. **c** Upon administration of tamoxifen in pregnant mice, Cre-recombinase is translocated in the nucleus of *Sox2*- or *Rax*-positive cells and deletes exon 3 of the *Ctnnb1* gene, resulting in a stabilized beta-Catenin protein. Simultaneously, SmoM2 is constitutively expressed. Consequently, both Wnt and Shh signaling are upregulated in *Sox2-creER*^*T2*^*::Ctnnb1(ex3)*^*fl/+*^*SmoM2*^*fl/+*^*(SBS)* and *Rax-creER*^*T2*^*::Ctnnb1(ex3)*^*fl/+*^*SmoM2*^*fl/+*^ *(RBS)* mice. **d**–**f** Overview H&E ocular histology of control (*Ctnnb1(ex3)*^*fl/+*^*SmoM2*^*fl/+*^, **d**) and mutant mice (*SBS*, **e**, and *RBS*, **f**) after Shh and Wnt co-activation initiated on E8.5. In *SBS* and *RBS* mice, ocular structures were obliterated and replaced by cellular infiltrates with periretinal dissemination. **g**–**i** High-magnification images of the prospective ciliary body region. Control eyes at day E18.5 displayed clearly distinguishable emerging inner ciliary epithelium and initiation of the folding of the outer ciliary epithelium (**g**). Corresponding regions in *SBS* (**h**) and *RBS* (**i**) mice showed pigmented (**h**, asterisk) and clear, sheet-like growing cells (**i**, white arrowheads) in proximity of residuals of the prospective ciliary body. Lens tissue is marked with an “x.” **j**–**l** High-magnification images of retinal and retina-like rosette structures. The control neonatal retina displayed five major distinguishable layers (**j**). NFL nerve fiber layer, GCL ganglion cell layer, IPL inner plexiform layer, INL inner nuclear layer, NBL neuroblast layer. *SBS* (**k**) and *RBS* (**l**) mice frequently showed rosette-forming retina-like cells with central lumina. Rosettes appeared either unilayered (**k**, black arrowhead) or multilayered/pseudostratified, resembling derivative NBL (**l**, black arrow). **m**–**u** Overview MAP2C immunostaining of ocular histology of control (**m**) and mutant (**n**, **o**) mice. In the control retina, a predominance of MAP2C positivity was displayed in the GCL (**m**, **p**, **s**) and no staining was detected in the prospective ciliary body (**p**). In *SBS* (**n**) and *RBS* (**o**) mice, sheet-like growing cells displayed strong MAP2C expression (**n**, **o**, **r**, **q**, **u**) and demonstrated periretinal infiltration (**o**, arrowheads). Pseudostratified NBL-like rosettes, much like the NBL of controls, appeared negative (**t**, **u**). Scale bar in **d**–**f** and **m**–**o** is 500 µm. Scale bar in **g**–**l** and **p**–**u** is 100 µm. **v** Tabular overview of experimental replicates and observed phenotypes. Not all eyes of *SBS* E12.5 and *RBS* E8.5 litters were subjected to histomorphological assessment. **w**, **x** Cell fate determination of *Sox2*-positive (**w**) and *Rax*-positive (**x**) precursor cells demonstrated in the eyes of E18.5 *Rax*-*creER*^*T2*^*::R26-lsl-RFP* mice after tamoxifen administration on day E8.5. Mapped cells were found within the prospective ciliary body as well as throughout the neural retina in a columnar arrangement. Single cells of the retinal pigmented epithelium appeared positive. Scale bar is 250 µm. **y** Heat map of SHH^1^ and WNT^2^ pathway-related proteins demonstrated robust abundance in *RBS* E8.5 lesions compared to *RBS* eyes after later injection time points (E10.5, E12.5, and E14.5) and control eyes. Distance method used was Pearson correlation; dendrogram drawing method used was average linkage. Selection of proteins was based on gene sets of the mouse Nanostring PanCancer pathways panel. Biological replicates for E8.5–E14.5 were *n* = 2 and for control eyes *n* = 8 (*n* = 2 for each time point of tamoxifen administration). Gray color = missing value.

SOX2 and RAX are transcription factors with essential functions for early and late stages of embryonic eye development and have been described as markers for retinal precursor cells [37, 38]. An overview of ocular developmental stages and concomitant *Sox2* and *Rax* expression is illustrated and described in Fig. 3a. In brief, *Sox2* is broadly expressed in multiple ocular and extraocular structures during embryonic and postembryonic stages, while *Rax* shows stronger confinement to the retina at early phases of ocular development.

For spatiotemporal control of Wnt and Shh signaling activation during embryonal eye development in vivo, we used a tamoxifen-inducible cre recombinase (*creER*^*T2*^*)* under control of either the *Sox2* or the *Rax* promoter (*Sox2-creER*^*T2*^ [11] and *Rax-creER*^*T2*^ [13], Fig. 3b, c). Respective mutants were crossed with mice harboring floxed alleles of the Wnt and Shh pathway components *Ctnnb1(ex3)*^*fl/fl*^ [15] and *SmoM2*^*fl/f*^ [12], respectively. Tamoxifen administration in pregnant mice bearing offspring with *Ctnnb1(ex3)*^*fl/+*^*SmoM2*^*fl/+*^ and either *Sox2-creER*^*T2*^ (*SBS*) or *Rax-creER*^*T2*^ (*RBS*, Fig. 3b, c) was initiated at E8.5, E10.5, E12.5, or E14.5, and embryos were collected for analysis on E18.5 (Fig. 3d–u and Supplementary Fig. 1).

### Early *Sox2* and *Rax* dependent co-activation of Shh and Wnt signaling during embryonic development leads to formation of analogous tumor-like ocular lesions

Tamoxifen injection on day E8.5 of embryonic eye development resulted in obliteration of anatomical integrity of the eye in *SBS* and *RBS* mice on E18.5. The retinal continuity was disrupted and the intraocular space was filled with cellular infiltrates, which also expanded beyond the perimeter of the ocular chambers (Fig. 3d–l). The ocular lesions displayed intralesional heterogeneity, with the majority of the cell masses consisting of sheet-like/structurelessly spread monomorphous cells with clear cytoplasm (Fig. 3i, white arrowhead). Additionally, crowded neuroblastic cell formations in an either multilayered, pseudostratified rosette or misfolded appearing pattern were frequently found (Fig. 3k, l). Moreover, we observed monolayered rosette formations (Fig. 3k, black arrowhead) as well as cohesive groups of epithelioid cells with prominent pigmentation (Fig. 3h, asterisk). All of these features were found in *SBS* and *RBS* E8.5 lesions with no appreciable differences. Induction at later time points than E8.5 led to strongly attenuated ocular phenotypes. Eyes of *SBS* mice were characterized by rosette-like or misfolded disorganization of retinal elements, reminiscent of retinal dysplasia (Supplementary Fig. 1a–f). In comparison, *RBS* ocular morphology displayed less distinctive retinal misfolding (Supplementary Fig. 1g–l). Importantly, sheet-like monomorphously growing cells with expansive periretinal dissemination were not observed in any mice injected later than E8.5 (Fig. 3v).

Ocular lesions, which originated after tamoxifen injection on day E8.5, were assessed for protein expression characteristics by immunostaining. We found strong and homogeneously distributed cytoplasmic signal of MAP2C primarily in the sheet-like growing cell layer, while rosettes and residuals of the neuroblastic layer showed only weak positivity and pigmented cell groups appeared negative (Fig. 3m–u). The neonatal control retina showed strong staining primarily in the ganglion cell layer (GCL), whereas the outer layers of the retina displayed only faint staining. Extensive immunostaining analysis demonstrated that *Sox2* and *Rax* promoter driven ocular lesions shared consistent protein expression characteristics (Supplementary Fig. 2). In order to detect the possible cell of origin for *SBS* and *RBS* ocular lesions, we characterized the fate of *Sox2*- and *Rax*-positive cells targeted at E8.5. We performed fate mapping experiments using a tamoxifen inducible RFP expressing mouse strain (*R26-lsl-RFP*) [14]. RFP staining revealed the targeted *Sox2*- and *Rax*-positive cells on E8.5 to comprise precursor cells of the prospective ciliary body as well as columnar distributed cells of all retina layers (Fig. 3w, x). Thus, we concluded that *SBS* and *RBS* ocular tissues displayed no apparent distinctions and represented analogous lesions.

### *RBS* E8.5 ocular lesions rely on time point-specific co-activation of Shh and Wnt and demonstrate marked differences in protein analyses compared to the developing eye

With regard to the strong retinal confinement of *Rax* promoter activity during development, we considered *RBS* to represent the favorable mouse model over *SBS* and continued with further investigations on the former. In order to explore the striking attenuation of *RBS* phenotypes after tamoxifen injection at later time points than E8.5, we subjected the ocular tissue of all *RBS* experimental groups to proteomic analysis. We found that Shh and Wnt pathway related proteins were highly abundant in E8.5 lesions compared to lesions generated after later injection time points and control eyes (Fig. 3y). Moreover, in line with the prevailing disseminated MAP2C-positive cells of E8.5 lesions, proteomic profiles strongly reflected immature neuronal signatures and similarity to murine ETMR-like embryonal tumors [8] (Supplementary Fig. 3).

Our subsequent experiments focused on *RBS* lesions after tamoxifen administration on E8.5. We first asked whether single pathway activation (either Shh or Wnt) at E8.5 may be sufficient to drive tumor-like lesions. We therefore performed tamoxifen injection on day E8.5 in *Rax*-*creER*^*T2*^*::Ctnnb1(ex3)*^*fl/+*^(*RB*) or *Rax*-*creER*^*T2*^*::SmoM2*^*fl/+*^ (*RS*) mice for sole activation of either the Wnt or the Shh pathway. We found no ocular phenotypes with periretinal expansion and hence no concordance to *RBS* lesions in these mice (Supplementary Fig. 4). Therefore, co-activation of both pathways, Shh and Wnt is crucial for the *RBS* (E8.5 induced) ocular phenotype.

**Fig. 4:**
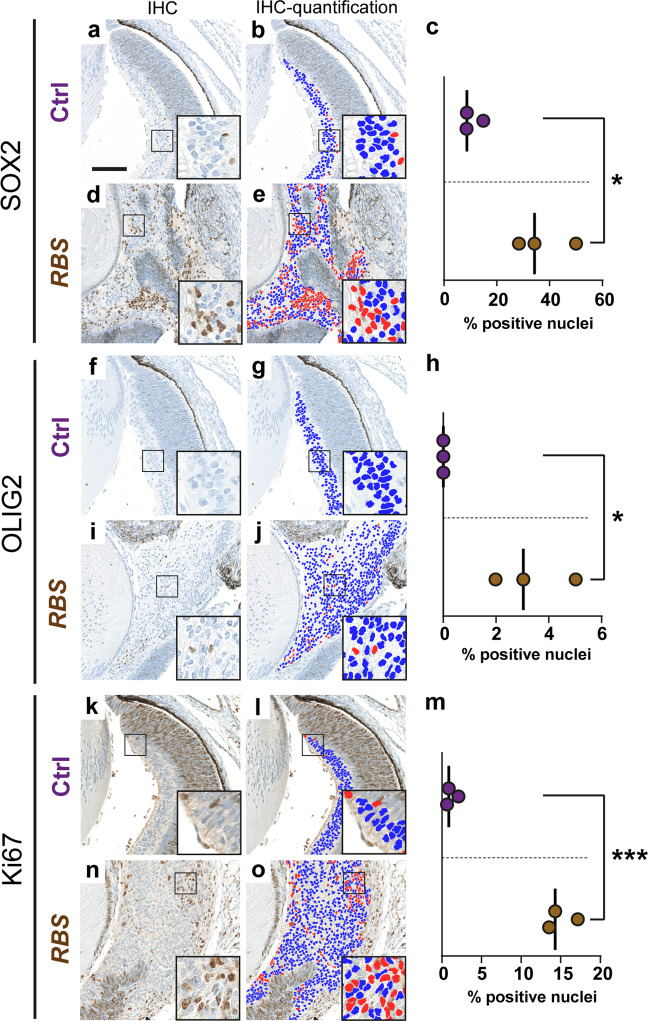
Quantification of immunohistochemical nuclear staining in control retina and *Rax*-*creER*^*T2*^*::Ctnnb1(ex3)*^*fl/+*^*SmoM2*^*fl/+*^ (*RBS*) ocular lesions. Digitally supported quantification of SOX2 (**a**–**e**), OLIG2 (**f**–**j**), and Ki67 (**k**–**o**) nuclear staining in inner retinal layers of control mice (**a**, **f**, **k**) and sheet-like growing masses of *RBS* E8.5 ocular lesions (**d**, **i**, **n**). Nuclear signal of SOX2 (**c**), OLIG (**h**), and Ki67 (**m**) was significantly increased in mutant *RBS* lesions (brown) compared to control retinas (purple). Negative nuclei are labeled blue, positive nuclei are labeled red (**b**, **e**, **g**, **j**, **l**, **o**). In **c**, **h**, **m**, lines indicate medians. Asterisks indicate significance: *p* value <0.05*, <0.001***, two-tailed *t* test. Scale bar is 100 µm.

Next, in order to assess immunohistochemical differences of *RBS* lesions compared to control retinas in an impartial way, we quantified immunostaining signals digitally using the Fiji/ImageJ software [28] The sheet-like growing cells of *RBS* lesions and the GCL of the control retina, which corresponded in morphology and MAP2C positivity, were designated as the cellular compartments eligible for quantification in both tissues (Fig. 4a–o).

For identification of quantifiable differences between mutant and control tissue, we evaluated nuclear staining signals for markers of the retina, such as SOX2 and OLIG2, and assessed proliferation via Ki67. In *RBS* lesions, SOX2 expression was strongly present in the majority of cells of the sheet-like growing compartment as well as moderately strong in the rosette elements (Supplementary Fig. 2e, f and Fig. 4d, e). Digitally supported quantification of nuclear signal in *RBS* lesions compared to control eyes demonstrated significant increase of SOX2-positive cells in *RBS* lesions compared to inner retinas (*p* = 0.0167, Fig. 4c). Additionally, OLIG2-positive cells were found in scattered distribution in the sheet-like growing compartment (Supplementary Fig. 2l and Fig. 4i, j). Digital quantification confirmed the increase of OLIG2-positive cells in *RBS* lesions (*p* = 0.0199, Fig. 4h). In order to assess proliferation, Ki67 staining was analyzed. In the control retina, single Ki67-positive cells were detected in the GCL (Supplementary Fig. 2y, z and Fig. 4k, l), whereas a significantly higher percentage of positive cells was detected in the sheet-like growing compartment of *RBS* lesions (*p* = 0.0003, Supplementary Fig. 2ac, ad and Fig. 4m, n).

In conclusion, co-activation of Shh and Wnt signaling in *Sox2*- and *Rax-*positive retinal precursor cells at E8.5 resulted in the formation of tumor-like ocular lesions. These comprised components of the immature retina with increase in the nuclear expression of SOX2 and OLIG2. Additionally, an increased Ki67 proliferation index was detected in the sheet-like growing compartment of *RBS* lesions.

### Histomorphology, immunohistochemistry as well as molecular profiles of *RBS* ocular lesions demonstrate similarity with human IO-MEPL

**Fig. 5:**
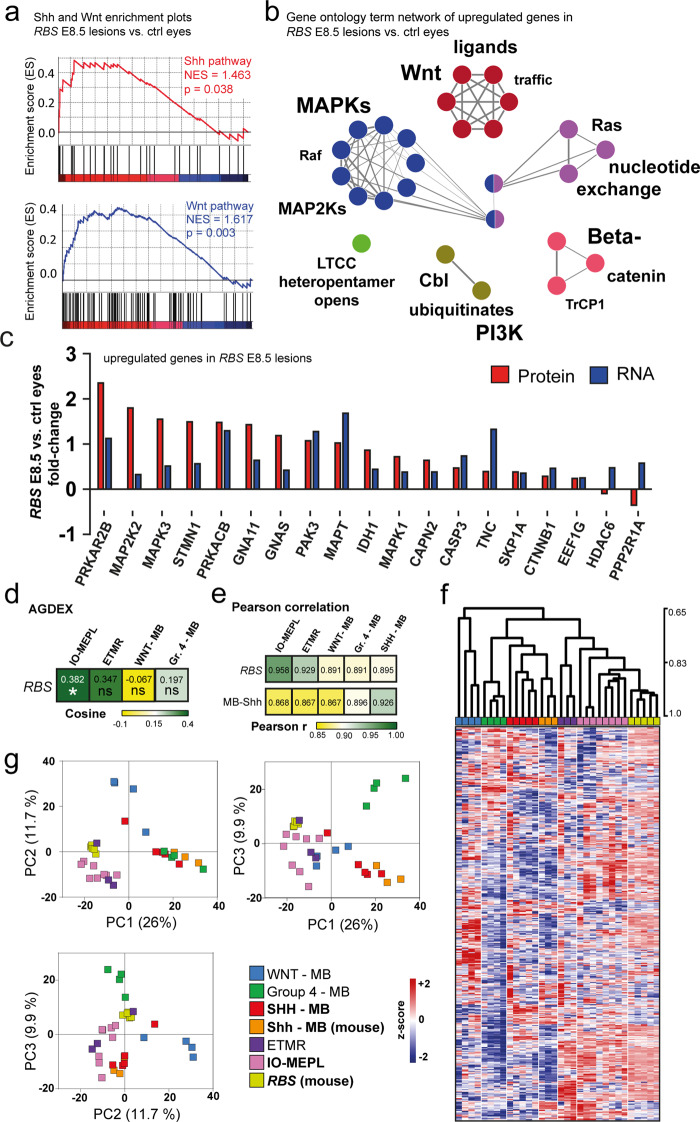
*Rax*-*creER*^*T2*^*::Ctnnb1(ex3)*^*fl/+*^*SmoM2*^*fl/+*^ (*RBS*) ocular lesions display molecular similarities to human IO-MEPL. **a** Both Shh and Wnt pathway genes were overrepresented in *RBS* ocular lesions in comparison to control eyes. GSEA was performed based on predefined Nanostring gene sets. **b** Functional gene ontology (GO) term network of upregulated genes in *RBS* lesions compared to control eyes. Each node represents a significant GO term. Node colors indicate affiliation of GO terms to GO groups. Node clusters are annotated with the top three most significant words of the respective GO term aggregation. Font size differences within the annotation of a distinct cluster represent varying significances. Edges indicate term–term inter-relations with Kappa score >0.3. Upregulated genes were determined by Welch-corrected *t* test with adjusted Bonferroni correction. **c** LC-MS/MS protein levels of genes identified as significantly upregulated in *RBS E8.5* vs. control eyes. **d** Agreement of differential expression (AGDEX) revealed significant agreement of *RBS* lesions and human IO-MEPLs. Mouse Shh-MBs and human SHH-MBs were taken as a reference. Asterisk indicates significance: *p* value <0.05*. **e** Pearson correlation analysis based on cross-species harmonized gene expression data showed highest correlation of *RBS* lesions with human IO-MEPL and highest correlation of mouse Shh-MBs with human SHH-MBs. **f** Hierarchical clustering (based on Pearson correlation) based on cross-species harmonized gene expression data. Ocular lesions in *RBS* mice formed a distinct cluster with human IO-MEPLs. Established Shh-MBs from 14-day-old *hGFAP-cre::SmoM2*^*fl/+*^ mice, which formed a distinct cluster together with SHH-MBs served as a reference control. Analysis incorporated the top 60% of genes with highest variance. **g** Principal component analysis (PCA) plots based on cross-species harmonized gene expression data showed species-independent grouping of samples. Analysis incorporated the top 60% of genes with highest variance. Cross-species gene expression analysis was based on 718 common genes of the human and mouse PanCancer pathways panels (Nanostring Technologies).

Next, we analyzed gene expression data of *RBS* ocular lesions (*n* = 5) and control eyes (*n* = 4). Murine Shh-MBs (*n* = 3) of *hGFAP-cre::SmoM2*^*fl/+*^ mice [39] were used as additional reference controls. Co-activation of Shh and Wnt signaling in *RBS* lesions compared to control eyes was confirmed using GSEA (NES_Shh_ = 1.46, *p* = 0.038, NES_Wnt_ = 1.617, *p* = 0.003, Fig. 5a). Moreover, a GO network based on significantly overexpressed genes in *RBS* lesions compared to control eyes demonstrated activation of Wnt pathway components as well as RAF and MAPK related terms (Fig. 5b and Supplementary Table 3). In contrast, upregulated genes of developing E18.5 control eyes compared to *RBS* lesions mainly attributed to DNA replication (Supplementary Fig. 5a and Supplementary Table 4). Of note, a significant subset of the genes of the Nanostring panel was also detected in proteomic analyses (Supplementary Fig. 5b). Protein measurements confirmed robust fold changes (Fig. 5c and Supplementary Fig 5c).

We next asked whether human IO-MEPLs and murine *RBS* lesions showed molecular similarities. Gene expression data of human tumors and murine lesions was utilized for an AGDEX analysis [26]. Established murine Shh-MBs as well as human SHH-MBs were chosen as a reference in each species. The results indicated highest (Cosine = 0.382) and significant (*p* = 0.036) agreement of *RBS* lesions to human IO-MEPLs among the tested samples (Fig. 5d). To overcome the sparsity of available and healthy analogous retinal reference tissue of humans and mice, we aligned human and murine gene expression data using ComBat [22] for additional cross-species analyses. Means and variations of biological conditions and high overlap of significant genes between tumor groups confirmed efficient ComBat-based data harmonization (Supplementary Fig. 6). Pearson correlation analysis of cross-species harmonized data revealed the highest similarity of gene expression profiles from *RBS* ocular lesions and human IO-MEPLs (Pearson *r* = 0.958, *p* < 0.001, Fig. 5e). As expected, murine Shh-MBs displayed the highest similarity to human SHH-MBs (Pearson *r* = 0.926, *p* < 0.001, Fig. 5e). Unsupervised hierarchical clustering (Fig. 5f) as well as principal component analysis (Fig. 5g) additionally confirmed molecular similarity of *RBS* lesions and IO-MEPLs in a comparable manner as was demonstrable for murine Shh-MBs and SHH-MBs.

**Fig. 6:**
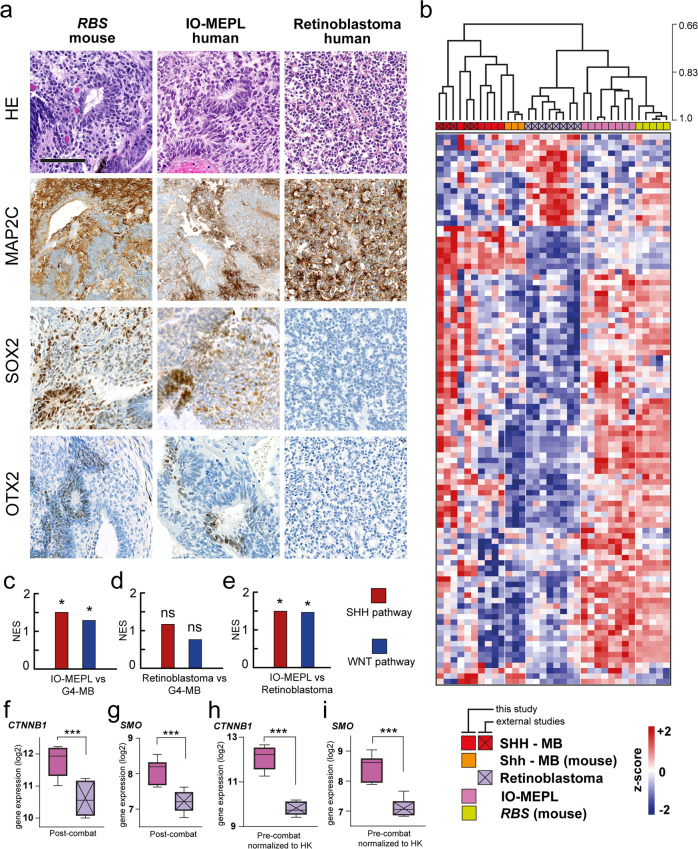
Morphological, immunohistochemical, and molecular features of *Rax*-*creER*^*T2*^*::Ctnnb1(ex3)*^*fl/+*^*SmoM2*^*fl/+*^ (*RBS*) ocular lesions match with IO-MEPL as opposed to retinoblastoma. **a** Morphology of murine *RBS* lesion after tamoxifen administration on day E8.5 (left column) compared to IO-MEPL (middle column) and human retinoblastoma (right column). Flexner–Wintersteiner–Rosettes in retinoblastoma were smaller in comparison to the pseudostratified rosettes of IO-MEPL and *RBS* (first row). IO-MEPL and *RBS* stained strongly for MAP2C, while rosette structures consistently lacked staining signal. In contrast, retinoblastoma displayed strong MAP2C positivity with most prominent staining intensity towards the luminal surfaces of rosettes (second row). SOX2-positive cells were found in IO-MEPL and *RBS* in a scattered distribution with occasional accentuation of staining intensity in association with rosettes or rosette-like structures, while SOX2 staining was negative in Retinoblastoma (third row). OTX2-positive cells were occasionally found in IO-MEPL in association with rosettes or rosette-like structures and in *RBS* in the residual retina and NBL-like rosettes. In contrast, retinoblastoma stained negative for OTX2 (fourth row). Scale bar is 100 µm. **b** Hierarchical clustering based on cross-species harmonized gene expression data of this study, GSE30074 [33] and GSE172170 [34]. Ocular lesions of *RBS* mice formed a distinct cluster with closest similarity to human IO-MEPLs, but not human retinoblastomas. The analysis was based on significantly differentially expressed genes of IO-MEPL vs. retinoblastoma (*t* test). Established Shh-MBs from *hGFAP-cre::SmoM2fl/+* mice formed a distinct cluster with SHH-MBs of this study and GSE30074. Cross-species gene expression analysis was based on 704 common genes of the human and mouse Nanostring PanCancer pathway panels as well as the Affymetrix Human Gene 1.0 ST Array [HuGene-1_0-st]. Distance method used was Pearson correlation, dendrogram drawing method used was average linkage. **c**–**e** Gene set enrichment analysis (GSEA) revealed persisting overrepresentation of both WNT and SHH signaling pathway genes in IO-MEPLs vs. G4-MB after data harmonization (**c**) and significant enrichment of both pathways in IO-MEPLs compared to retinoblastoma (**d**, **e**). Analysis was performed based on predefined Nanostring gene sets. Asterisks indicate significance: *p* value <0.05*. **f**–**i** Box plots demonstrate significantly upregulation of both *CTNNB1* and *SMO* in IO-MEPL compared to retinoblastoma. Gene expression comparisons were based on the datasets after Combat harmonization (**f**, **g**) and on the original datasets normalized to housekeeping genes (HK, **h**, **i**). Whiskers extend from min to max values. Asterisks indicate significance: *p* value <0.001***, Welch’s *t* test.

Since retinoblastoma represents the most common pediatric ocular neoplasm and a potential differential diagnosis to IO-MEPL, we aimed to compare retinoblastoma characteristics with features of murine *RBS* lesions and human IO-MEPL. For this, we investigated histomorphology and immunostaining patterns in representative cases of these lesions. IO-MEPL showed strong concordance concerning rosette morphology and distribution of MAP2C-, SOX2- and OTX2-positive cells compared to *RBS* lesions. In contrast, typical Flexner–Wintersteiner–Rosettes of retinoblastoma displayed highly divergent morphological features and immunostaining patterns (Fig. 6a). Next, we utilized publicly available gene expression data of retinoblastomas [34], to compare with our obtained gene expression data of *RBS* lesions and IO-MEPLs (Fig. 6b–i and Supplementary Fig. 7). We found that similarities in gene expression profiles remained consistent between *RBS* lesions and IO-MEPLs over retinoblastomas (Fig. 6b). Also, SHH and WNT pathways were significantly enriched in IO-MEPLs compared to retinoblastomas (Fig. 6c–e) with increased expression levels of *CTNNB1* and *SMO* (Fig. 6f–i). Conclusively, murine *RBS* ocular lesions were similar to human IO-MEPL based on histomorphology, immunohistochemistry, and gene expression profiles.

In summary, we outline Shh and Wnt co-activation as a molecular feature of IO-MEPLs referenced by ETMR and presented a promising novel model system in murine embryogenesis recapitulating features of human IO-MEPL.

## Discussion

This study is the first to analyze and present RNA expression data obtained from IO-MEPLs. We describe strong congruence of IO-MEPLs and ETMR expression profiles regarding SHH and WNT signaling. While the roles of both these pathways have been previously established in ETMR molecular biology [8, 40], this represents a novel finding for IO-MEPLs. Our results are particularly interesting, since previous studies have suggested a differing origin of ETMR and IO-MEPL, as demonstrated by inconsistent DNA methylation profiles [7] and the lack of C19q13.42 alterations in the latter [5]. Moreover, generic genomic alterations in IO-MEPL, including mutations of *DICER1* and *KMT2D*, have been described [9]. Since a more recent report has characterized ETMRs with *DICER1* mutations, which also lack C19MC amplification [40], one might speculate about a common driving mechanism in IO-MEPLs and a subset of ETMR. Of note, our *RBS* lesions also displayed similarity to murine ETMR-like tumors [8] based on global proteomic analyses. Conclusive clarification regarding the relationship of IO-MEPL and ETMR remains pending.

In an experimental mouse model, we showed that ocular Shh and Wnt co-activation during embryonal development is sufficient to drive tumor-like ocular lesions. Wnt and Shh signaling pathways serve physiological functions during eye development [41]. Intriguingly, both *Ctnnb1* and *Smo* appear to be essential for the proper formation of the optic cup during early oculogenesis [42, 43]. Conversely, Shh activation has been suggested to stimulate proliferation of retinal precursor cells in various organisms [44] and overexpression of stabilized *Ctnnb1* has been shown to lead to an expansion of the ciliary margin in mice [45]. However, and to our knowledge, no previously described mouse models involving dysregulation of Shh or Wnt signaling have been reported to generate ocular tumor-like lesions. This may relate to our finding that activation of both Shh and Wnt were crucial for the occurrence of tumor-like lesions with periretinal expansion. Given that previous studies have suggested Shh signaling to negatively regulate Wnt activity in the context of malignancies [46], our model may rely on bypassing this effect.

The time point of co-activation initiation on E8.5 was also a key factor in our mouse model. This is likely due to essential functions of Shh and Wnt for the formation of the optic vesicle and optic cup during early eye development and underlines the narrow temporal window of opportunity for Shh and Wnt signaling to majorly impact on distribution and expansion of targeted susceptible cells. Correspondingly, protein analyses demonstrated strongly attenuated Shh and Wnt co-activation when initiation occurred at time points later than E8.5. Of note, since *Sox2* expression is more broadly distributed compared to *Rax* [11, 37], one might assume that co-activation regulated by the *Sox2*-promoter may target a larger number of retinal precursor cells at stages of development. Thus, co-activation initiated later than E8.5 might call forth more widespread regulatory disruption by Shh and Wnt overactivation and lead to more prominent disturbances of retinal layering in the *SBS*- compared to the *RBS-*based model.

The *RBS* mouse model system we report in this study demonstrated a robust and severe ocular phenotype and confirmable targeted co-activation of Shh and Wnt in early retinal precursor cells. It is important to note that the conjecture of malignant features in *RBS* lesions is primarily rested on the morphological assessment of infiltrative behavior and concomitant destruction of ocular tissue integrity. Side effects of tamoxifen administration during early gestation [47] prohibited postnatal analyses of *RBS* lesions.

Modeling of human ocular neoplasms in mice is generally challenged by marked species-related genetic and cellular differences related to the eye compartment, as it has been previously highlighted in retinoblastoma research [48, 49]. In contrast to human ocular biology, loss of Rb1 is not sufficient to generate retinoblastomas in mice [50], and thus, similar underlying genetic differences may explain why loss of Dicer1 functions have not yet been associated with IO-MEPL-like lesions in previous mouse studies [51]. While morphological, immunohistochemical and molecular resemblance of *RBS* lesions and IO-MEPLs was striking in our investigations—and did not comparably correspond to retinoblastomas—we conclude that awareness for challenges in cross-species comparisons of eye lesions is warranted.

In summary, we have demonstrated Shh and Wnt signaling as a molecular feature of IO-MEPLs and described the occurrence of tumor-like lesions with features of IO-MEPLs upon co-activation of Shh and Wnt signaling during embryonal retinal development. Finally, our results may provide the basis for future studies investigating targeted therapeutic options in patients diagnosed with IO-MEPL.

## Supplementary information

Suppl Figure 1

Suppl Figure 2

Suppl Figure 3

Suppl Figure 4

Suppl Figure 5

Suppl Figure 6

Suppl Figure 7

Supplementary Table 1

Supplementary Table 2

Supplementary Table 3

Supplementary Table 4

Supplementary Table 5

authorship agreement form
