# Supplementary material for: Co-activation of Sonic hedgehog and Wnt signaling in murine retinal precursor cells drives ocular lesions with features of intraocular medulloepithelioma
Source: Oncogenesis. 2021 Nov 16;10(11):78. doi: 10.1038/s41389-021-00369-0 (PMC8595639; doi:10.1038/s41389-021-00369-0)
Supplement: Supplementary file 1 — Suppl Figure 1 [file 41389_2021_369_MOESM1_ESM.pdf]

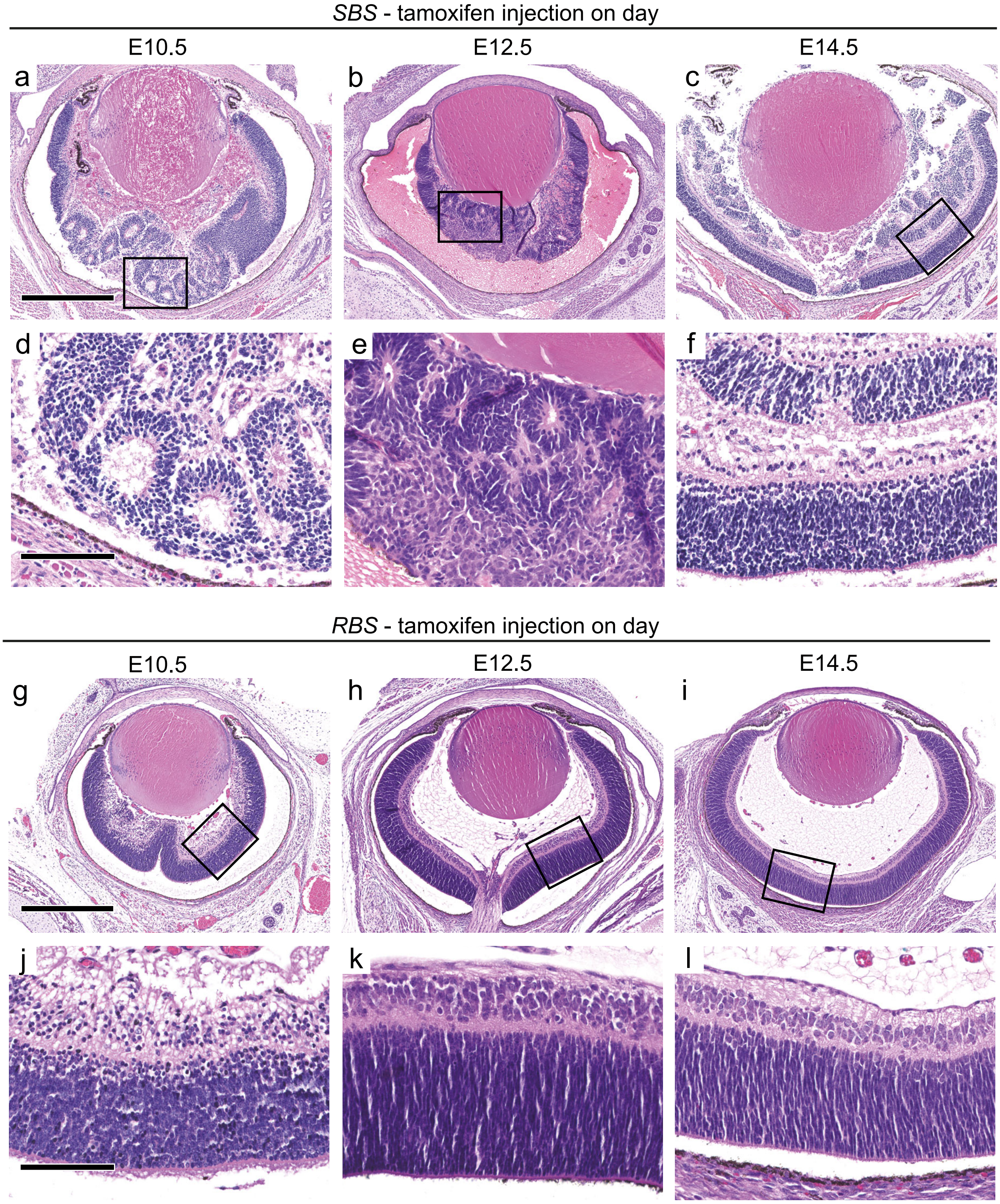

Supplementary Figure 1: Ocular phenotype of *Sox2-creER^{T2}::Ctnnb1(ex3)$^{fl/+}$SmoM2$^{fl/+}$* (*SBS*) and *Rax-creER^{T2}::Ctnnb1(ex3)$^{fl/+}$SmoM2$^{fl/+}$* (*RBS*) mice after tamoxifen administration on day E10.5, E12.5 and E14.5

a – f) Histologic overview (a - c) and high magnification images (d - f) of eyes of *SBS* mice after tamoxifen injection at time points E10.5, E12.5 and E14.5. Shh and Wnt activation at E10.5 and E12.5 resulted in disrupted retinal layering and rosette formation (a, d, b, e). After injection on E14.5, changes in retinal morphology were attenuated with a predominantly misfolded appearance (c, f).

g – l) Histologic overview (g-i) and high magnification images (j-l) of eyes of *RBS* mice after tamoxifen injection at time points E10.5, E12.5 and E14.5. Shh and Wnt activation at E10.5 (g, j) resulted in mild retinal misfolding and dispersion of the GCL. Induction at later time points E12.5 and E14.5 did not result in obvious changes of eye histomorphology (h, k, i, l).

Scale bar in a - c and g - i is 500µm. Scale bar in d – f and j – l is 100µm.
