# Supplementary material for: Co-activation of Sonic hedgehog and Wnt signaling in murine retinal precursor cells drives ocular lesions with features of intraocular medulloepithelioma
Source: Oncogenesis. 2021 Nov 16;10(11):78. doi: 10.1038/s41389-021-00369-0 (PMC8595639; doi:10.1038/s41389-021-00369-0)
Supplement: Supplementary file 2 — Suppl Figure 2 [file 41389_2021_369_MOESM2_ESM.pdf]

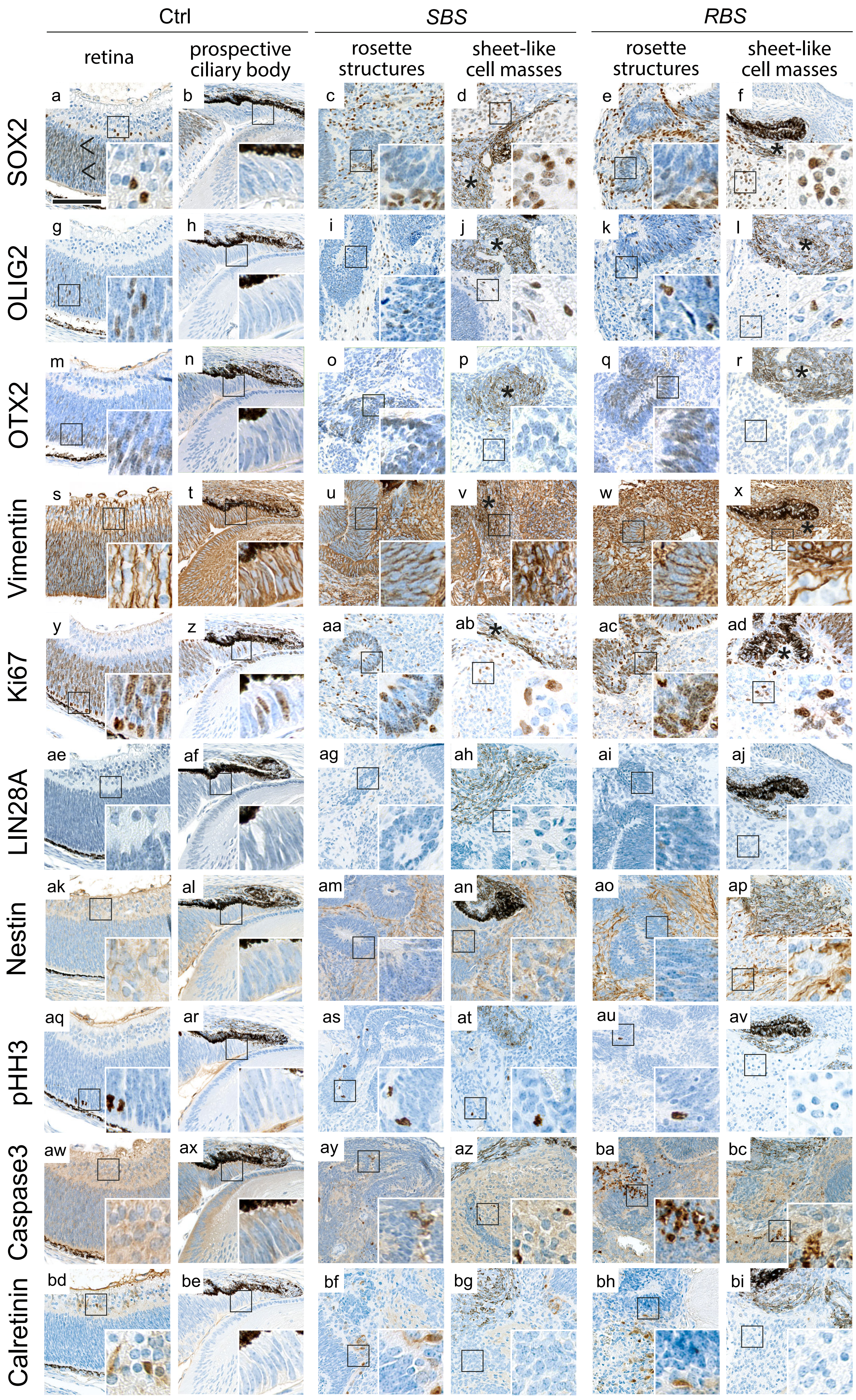
Ctrl
SBS
RBS
retina
prospective ciliary body
rosette structures
sheet-like cell masses
rosette structures
sheet-like cell masses
SOX2
OLIG2
OTX2
Vimentin
Ki67
LIN28A
Nestin
pHH3
Caspase3
Calretinin

Supplementary Figure 2: Immunohistochemical staining of *SBS* and *RBS* ocular lesions after tamoxifen administration on day E8.5

a - f) In controls, SOX2-positive cells were mainly found in the NBL (a, arrowheads) with only few SOX2-positive cells in the outer part of the GCL adjacent to the IPL. SOX2 was not detected in the prospective ciliary body (b). Increased SOX2-positivity is demonstrated in lesions of *SBS* (c, d) and *RBS* (e, f) mice, where scattered positive cells are found. SOX2-positive cells are also detected in the basal area of NBL-like rosettes (c, e).
g - l) OLIG2-positive cells were exclusively found throughout the NBL in the control retina (g, h). *SBS* (i, j) and *RBS* mice (k, l) displayed scattered OLIG2-positive cells within the NBL-like rosettes (i, k) and, to a fewer extend, in the sheet-like growing cell masses (j, l).
m - r) OTX2-positive cells were exclusively found in the outer layer of the NBL in the control retina (m, n). The NBL-like rosettes in lesions of *SBS* (o) and *RBS* mice (q) displayed a positive staining pattern near the inner luminal surface. In contrast, OTX2 was not detected in sheet-like growing cell masses (p, r).
s - x) Vimentin was strongly expressed throughout the retina and prospective ciliary body of the control eye (s, t) as well as throughout the eye lesions of *SBS* (u, v) and *RBS* mice (w, x).
y – ad) In controls, Ki67-positive cell nuclei are predominantly and densely represented in the NBL (y) and, to a fewer extend, in the GCL as well as the prospective ciliary body (z). The NBL-like rosettes in lesions of *SBS* (aa) and *RBS* mice (ac) showed dense Ki67 nuclear positivity. Additionally, Ki67-positive nuclei were frequently present in sheet-like cell masses (ab, ad).
ae – aj) The entire control retina (ae, af) as well as lesions of *SBS* (ag, ah) and *RBS* mice (ai, aj) were void of LIN28A staining.
ak – ap) Nestin staining was found predominantly in the GCL of control retinas (ak, al) and the sheet-like masses of *SBS* (an) and *RBS* (ap) lesions.
aq – av) In control retinas, pHH3-labeled mitosis figures were predominantly and occasionally found in the outer layer of the NBL. In lesions of RBS and SBS mice, pHH3-labeled mitosis figures were mainly found near the inner luminal surface of NBL-like rosettes (as, au) and occasionally in sheet-like cell masses (at).
aw – bc) Strong positivity for Caspase-3 was usually absent in the control retina (aw, ax). Strong caspase-3 staining was more frequently found throughout *SBS* (ay, az) and *RBS* (ba, bb) mice lesions with variable distribution and intensity.
bd – bi) Calretinin staining was found in the GCL of the control retina (bd). In *SBS* (bf) and *RBS* mice (bh) positivity for calretinin was predominantly found in regions best corresponding to remnants of the regular retina.
GCL= ganglion cell layer, IPL= inner plexiform layer, NBL= neuroblast layer. Scale bar is 100µm.
