# Supplementary material for: Co-activation of Sonic hedgehog and Wnt signaling in murine retinal precursor cells drives ocular lesions with features of intraocular medulloepithelioma
Source: Oncogenesis. 2021 Nov 16;10(11):78. doi: 10.1038/s41389-021-00369-0 (PMC8595639; doi:10.1038/s41389-021-00369-0)
Supplement: Supplementary file 3 — Suppl Figure 3 [file 41389_2021_369_MOESM3_ESM.pdf]

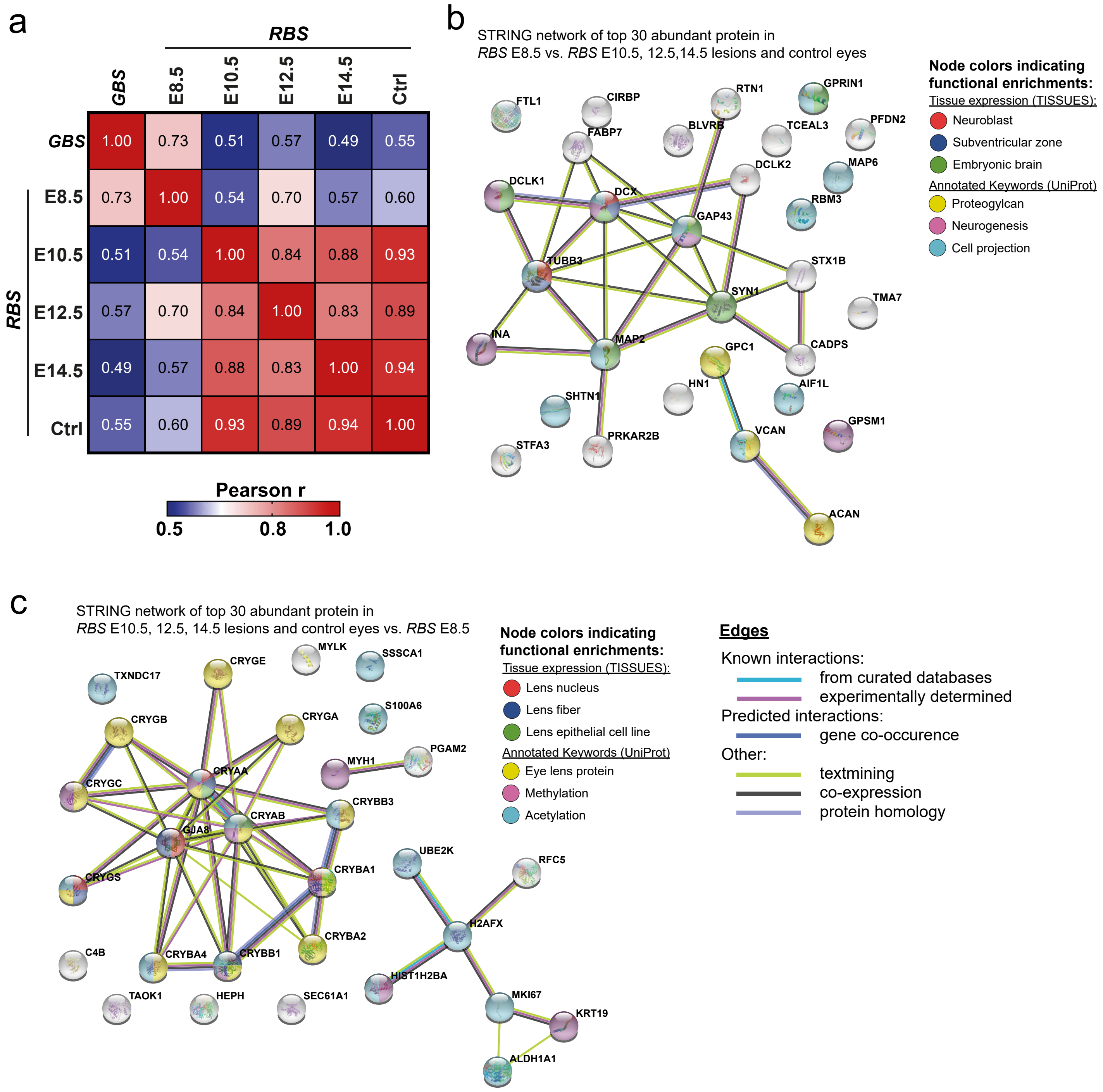

Supplementary Figure 3: *RBS* E8.5 lesions show distinct proteomic profiles and demonstrate similarity with ETMR-like murine embryonal brain tumors of E18.5 *hGFAP-cre::Ctnnb1(ex3)$^{fl/+}$SmoM2$^{fl/+}$* (*GBS*) mice

a) Correlation matrix of previously published murine ETMR-like *GBS* tumors (8) and *RBS* eyes after different tamoxifen injection time points. *RBS* E8.5 lesions showed stronger correlation with *GBS* tumors compared to *RBS* eyes after later injection time points and control eyes. Matrix is based on Pearson correlation of the top 100 variant proteins.

b - c) STRING connectivity network of the top 30 differentially abundant proteins of *RBS* E8.5 lesions vs. *RBS* 10.5, 12.5, 14.5 and control eyes (b) and vice versa (c). Network nodes represent proteins. Edges represent protein-protein associations. Colored nodes represent proteins affiliated with the top 3 strongest functional enrichments of the tissue expression database (TISSUES) (58) and annotated keywords of the Universal Protein Resource (UniProt) (59). Networks were generated via default parameters (full string network; medium confidence score 0.4; medium FDR stringency 5%) of STRING (Version 11.5) (ref. 60).
