# Supplementary material for: Co-activation of Sonic hedgehog and Wnt signaling in murine retinal precursor cells drives ocular lesions with features of intraocular medulloepithelioma
Source: Oncogenesis. 2021 Nov 16;10(11):78. doi: 10.1038/s41389-021-00369-0 (PMC8595639; doi:10.1038/s41389-021-00369-0)
Supplement: Supplementary file 4 — Suppl Figure 4 [file 41389_2021_369_MOESM4_ESM.pdf]

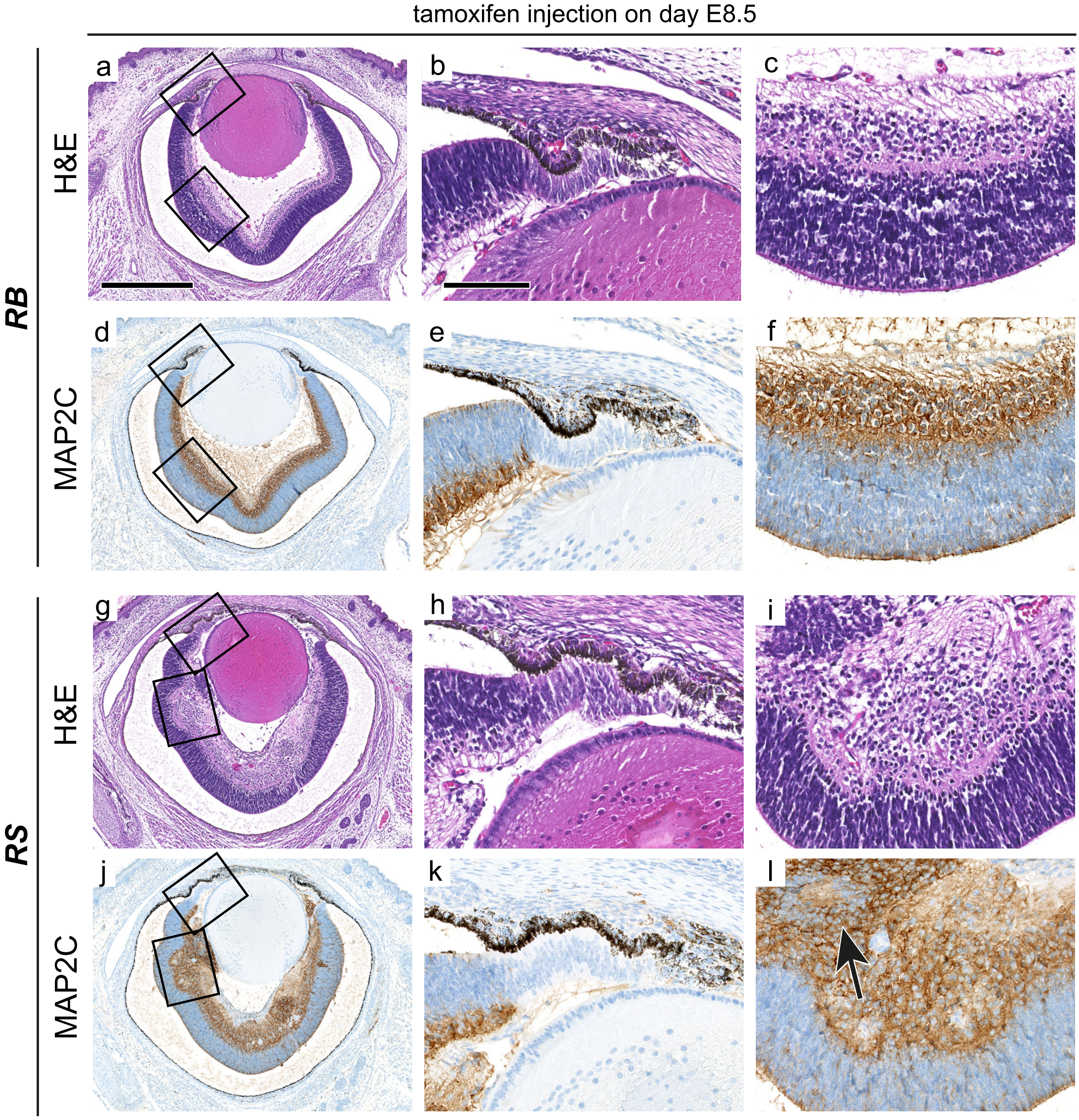

m

mouse strain	injection time point	n (animals investigated)	% cre + animals	no. of cre+ eyes investigated	no. of cre + eyes with expansive periretinal dissemination	disruption of retinal layering
RB	E8.5	10	70	7	0	7
RS	E8.5	4	75	6	0	6

Supplementary Figure 4: Ocular phenotype of *Rax-creER^{T2}::Ctnnb1(ex3)$^{fl/+}$*(*RB*) and *Rax-creER^{T2}::SmoM2$^{fl/+}$* (*RS*) E18.5 mice after tamoxifen administration on day E8.5

H&E ocular histology (a-c, g-i) and MAP2C staining (d-f, j-l) of *RB* (a-f) and *RS* (g-l) after sole Wnt or Shh activation initiated on E8.5. In the eyes of both *RB* and *RS,* structural inhomogeneities and dispersions of the retinal layers with occasional rosette formation (arrow) were visible. The phenotype appeared pronounced in *RS* mice. Expansive periretinal dissemination was not encountered (m). Not all eyes of *RB* E8.5 were subjected to histomorphological assessment. Scale bar in left column is 500 µm. Scale bar in middle and right column is 100 µm.
