# Supplementary material for: Co-activation of Sonic hedgehog and Wnt signaling in murine retinal precursor cells drives ocular lesions with features of intraocular medulloepithelioma
Source: Oncogenesis. 2021 Nov 16;10(11):78. doi: 10.1038/s41389-021-00369-0 (PMC8595639; doi:10.1038/s41389-021-00369-0)
Supplement: Supplementary file 5 — Suppl Figure 5 [file 41389_2021_369_MOESM5_ESM.pdf]

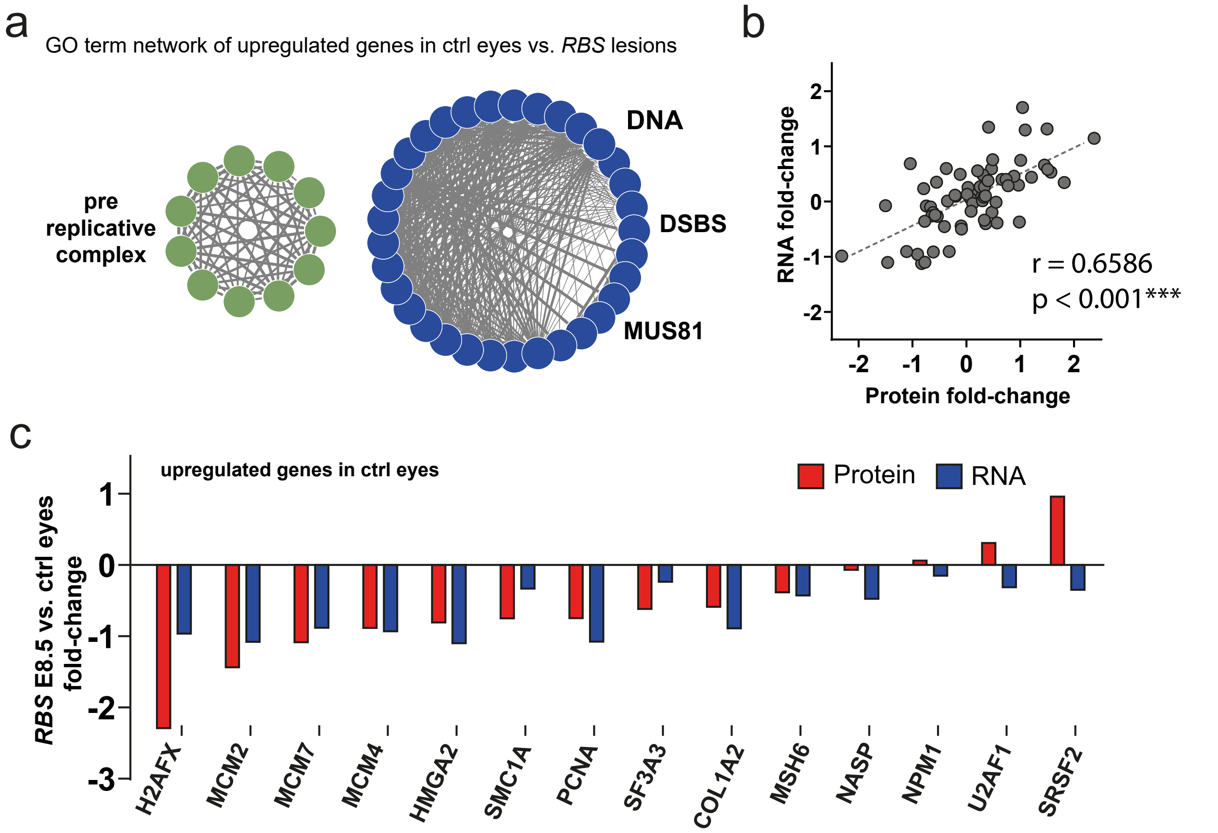

Supplementary Figure 5: Analyses of increased gene expression and protein abundancies in control eyes vs. *RBS* lesions

a) Gene ontology network of upregulated genes in control eyes. Each node represents a significant GO term. Node colors indicate affiliation of GO terms to GO groups. Node clusters are annotated with the top 3 most significant words of the respective GO term aggregation. Font size differences within the annotation of a distinct cluster represent varying significances. Edges indicate term-term interrelations with Kappa score > 0.3. Upregulated genes were determined by Welch-corrected t-test with adjusted Bonferroni correction.
b) A total of 66 matching genes and proteins were found in the Nanostring panel and proteomic analyses. Pearson correlation of RNA and protein fold-changes in *RBS* E8.5 vs control eyes was highly significant with $p<0.001$*** and r= 0.6586.
c) Protein levels of genes identified as significantly upregulated in *RBS E8.5* vs. control eyes.
