# Supplementary material for: Co-activation of Sonic hedgehog and Wnt signaling in murine retinal precursor cells drives ocular lesions with features of intraocular medulloepithelioma
Source: Oncogenesis. 2021 Nov 16;10(11):78. doi: 10.1038/s41389-021-00369-0 (PMC8595639; doi:10.1038/s41389-021-00369-0)
Supplement: Supplementary file 6 — Suppl Figure 6 [file 41389_2021_369_MOESM6_ESM.pdf]

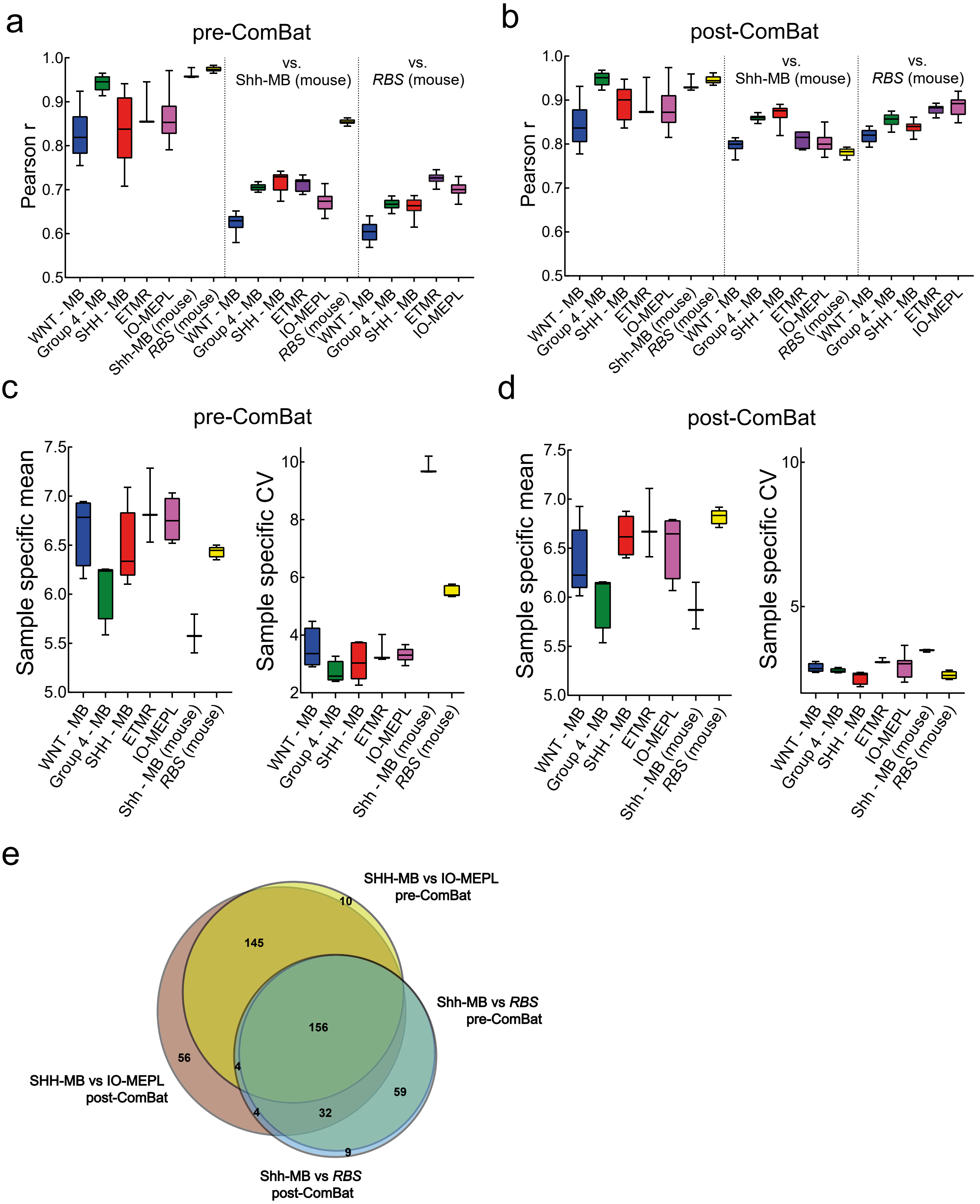

Supplementary Figure 6: ComBat based cross-species data harmonization

a - b) Pair wise Pearson correlation coefficient within each tumor entity and between human and murine lesions prior to (a) and after (b) ComBat based inter-species data harmonization.

c - d) Sample specific mean and coefficient of variation (CV) for each lesion prior to (c) and after (d) ComBat. Whiskers extend from min to max values in a – d.

e) Venn diagram visualization of t-test significant genes (p-value < 0.05; fold change > 2) between *RBS* and Shh-MB and IO-MEPL and SHH-MB.
