# Supplementary material for: Co-activation of Sonic hedgehog and Wnt signaling in murine retinal precursor cells drives ocular lesions with features of intraocular medulloepithelioma
Source: Oncogenesis. 2021 Nov 16;10(11):78. doi: 10.1038/s41389-021-00369-0 (PMC8595639; doi:10.1038/s41389-021-00369-0)
Supplement: Supplementary file 7 — Suppl Figure 7 [file 41389_2021_369_MOESM7_ESM.pdf]

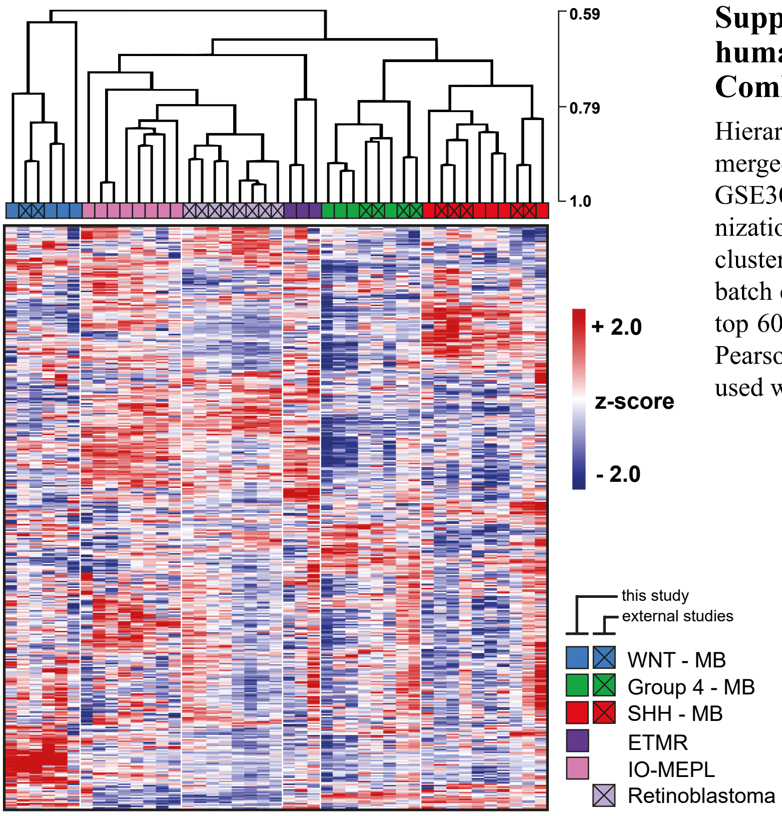

Supplementary Figure 7: Clustering of human embryonal tumors after ComBat based data harmonization

Hierarchical clustering of all human tumors of the merged gene expression data of this study and GSE30074 and GSE172170 after ComBat harmonization. Matching tumor entities homogenously clustered together demonstrating the efficiency of batch effect adjustment. Analysis was based on the top 60% variant genes. Distance method used was Pearson correlation, dendrogram drawing method used was average linkage.
