# Supplementary material for: Co-activation of Sonic hedgehog and Wnt signaling in murine retinal precursor cells drives ocular lesions with features of intraocular medulloepithelioma
Source: Oncogenesis. 2021 Nov 16;10(11):78. doi: 10.1038/s41389-021-00369-0 (PMC8595639; doi:10.1038/s41389-021-00369-0)
Supplement: Supplementary file 13 — authorship agreement form [file 41389_2021_369_MOESM13_ESM.pdf]

SPRINGER NATURE

In accordance with Springer Nature Authorship Policy we agree to change the authors of the manuscript as indicated below.

NAME OF JOURNAL: Oncogenesis

TITLE OF MANUSCRIPT: Co-activation of Sonic hedgehog and Wnt signaling in murine retinal precursor cells drives ocular lesions with features of intraocular medulloepithelioma

MANUSCRIPT NUMBER: MS#ONCSIS-21-0275-T

CORRESPONDING AUTHORS NAME: Prof. Dr. Julia E. Neumann

PREVIOUS AUTHOR NAMES:

Matthias Dottermusch*, Piotr Sumislawski*, Julia Krevet, Maximilian Middelkamp, Hannah Voß, Harald Bartsch, Karl Sotlar, Andrey Korshunov, Markus Glatzel, Ulrich Schüller, Julia E. Neumann

UPDATED AUTHOR NAMES:

Matthias Dottermusch*, Piotr Sumislawski*, Julia Krevet, Maximilian Middelkamp, Hannah Voß, Bente Siebels, Harald Bartsch, Karl Sotlar, Peter Meyer, Stephan Frank, Andrey Korshunov, Markus Glatzel, Ulrich Schüller, Julia E. Neumann

CHANGE TO AUTHOR LIST:

addition of 3 coauthors: Bente Siebels,Peter Meyer, Stephan Frank

Print Name	Signature	Date
Julia E. Neumann	[signature]	14/10/2021
Matthias Dottermusch	[signature]	14/10/2021

SPRINGER NATURE

In accordance with Springer Nature Authorship Policy we agree to change the authors of the manuscript as indicated below.

NAME OF JOURNAL: Oncogenesis

TITLE OF MANUSCRIPT: Co-activation of Sonic hedgehog and Wnt signaling in murine retinal precursor cells drives ocular lesions with features of intraocular medulloepithelioma

MANUSCRIPT NUMBER: MS#ONCSIS-21-0275-T

CORRESPONDING AUTHORS NAME: Prof. Dr. Julia E. Neumann

PREVIOUS AUTHOR NAMES:

Matthias Dottermusch*, Piotr Sumislawski*, Julia Krevet, Maximilian Middelkamp, Hannah Voß, Harald Bartsch, Karl Sotlar, Andrey Korshunov, Markus Glatzel, Ulrich Schüller, Julia E. Neumann

UPDATED AUTHOR NAMES:

Matthias Dottermusch*, Piotr Sumislawski*, Julia Krevet, Maximilian Middelkamp, Hannah Voß, Bente Siebels, Harald Bartsch, Karl Sotlar, Peter Meyer, Stephan Frank, Andrey Korshunov, Markus Glatzel, Ulrich Schüller, Julia E. Neumann

CHANGE TO AUTHOR LIST:

addition of 3 coauthors: Bente Siebels,Peter Meyer, Stephan Frank

Print Name	Signature	Date
PIOTR SUMISLAWSKI	P Sumislawski	03.10.2021

SPRINGER NATURE

In accordance with Springer Nature Authorship Policy we agree to change the authors of the manuscript as indicated below.

NAME OF JOURNAL: Oncogenesis

TITLE OF MANUSCRIPT: Co-activation of Sonic hedgehog and Wnt signaling in murine retinal precursor cells drives ocular lesions with features of intraocular medulloepithelioma

MANUSCRIPT NUMBER: MS#ONCSIS-21-0275-T

CORRESPONDING AUTHORS NAME: Prof. Dr. Julia E. Neumann

PREVIOUS AUTHOR NAMES:

Matthias Dottermusch*, Piotr Sumislawski*, Julia Krevet, Maximilian Middelkamp, Hannah Voß, Harald Bartsch, Karl Sotlar, Andrey Korshunov, Markus Glatzel, Ulrich Schüller, Julia E. Neumann

UPDATED AUTHOR NAMES:

Matthias Dottermusch*, Piotr Sumislawski*, Julia Krevet, Maximilian Middelkamp, Hannah Voß, Bente Siebels, Harald Bartsch, Karl Sotlar, Peter Meyer, Stephan Frank, Andrey Korshunov, Markus Glatzel, Ulrich Schüller, Julia E. Neumann

CHANGE TO AUTHOR LIST:

addition of 3 coauthors: Bente Siebels,Peter Meyer, Stephan Frank

Print Name	Signature	Date
Maximilian Middelkamp	[signature]	03.10.2021

SPRINGER NATURE

In accordance with Springer Nature Authorship Policy we agree to change the authors of the manuscript as indicated below.

NAME OF JOURNAL: Oncogenesis

TITLE OF MANUSCRIPT: Co-activation of Sonic hedgehog and Wnt signaling in murine retinal precursor cells drives ocular lesions with features of intraocular medulloepithelioma

MANUSCRIPT NUMBER: MS#ONCSIS-21-0275-T

CORRESPONDING AUTHORS NAME: Prof. Dr. Julia E. Neumann

PREVIOUS AUTHOR NAMES:

Matthias Dottermusch*, Piotr Sumislawski*, Julia Krevet, Maximilian Middelkamp, Hannah Voß, Harald Bartsch, Karl Sotlar, Andrey Korshunov, Markus Glatzel, Ulrich Schüller, Julia E. Neumann

UPDATED AUTHOR NAMES:

Matthias Dottermusch*, Piotr Sumislawski*, Julia Krevet, Maximilian Middelkamp, Hannah Voß, Bente Siebels, Harald Bartsch, Karl Sotlar, Peter Meyer, Stephan Frank, Andrey Korshunov, Markus Glatzel, Ulrich Schüller, Julia E. Neumann

CHANGE TO AUTHOR LIST:

addition of 3 coauthors: Bente Siebels,Peter Meyer, Stephan Frank

Print Name	Signature	Date
Krevet, Julia	[signature]	06.10.2021

UTHORS NAME: Prof. Dr. Julia E. Neuman

NAMES:

sch*, Piotr Sumislawski*, Julia Krevet, Maximilian Midd
ld Bartsch, Karl Sotlar, Andrey Korshunov, Markus Gl
leumann

IAMES:

sch*, Piotr Sumislawski*, Julia Krevet, Maximilian Midd
e Siebels, Harald Bartsch, Karl Sotlar, Peter Meyer, St
, Markus Glatzel, Ulrich Schüller, Julia E. Neumann

R LIST:

oauthors: Bente Siebels,Peter Meyer, St

	Signature	Dat
ls	Sie	03

SPRINGER NATURE

In accordance with Springer Nature Authorship Policy we agree to change the authors of the manuscript as indicated below.

NAME OF JOURNAL: Oncogenesis

TITLE OF MANUSCRIPT: Co-activation of Sonic hedgehog and Wnt signaling in murine retinal precursor cells drives ocular lesions with features of intraocular medulloepithelioma

MANUSCRIPT NUMBER: MS#ONCSIS-21-0275-T

CORRESPONDING AUTHORS NAME: Prof. Dr. Julia E. Neumann

PREVIOUS AUTHOR NAMES:

Matthias Dottermusch*, Piotr Sumislawski*, Julia Krevet, Maximilian Middelkamp, Hannah Voß, Harald Bartsch, Karl Sotlar, Andrey Korshunov, Markus Glatzel, Ulrich Schüller, Julia E. Neumann

UPDATED AUTHOR NAMES:

Matthias Dottermusch*, Piotr Sumislawski*, Julia Krevet, Maximilian Middelkamp, Hannah Voß, Bente Siebels, Harald Bartsch, Karl Sotlar, Peter Meyer, Stephan Frank, Andrey Korshunov, Markus Glatzel, Ulrich Schüller, Julia E. Neumann

CHANGE TO AUTHOR LIST:

addition of 3 coauthors: Bente Siebels,Peter Meyer, Stephan Frank

Print Name	Signature	Date
Meyer Peter	P. Meyer	3.10.2021

SPRINGER NATURE

In accordance with Springer Nature Authorship Policy we agree to change the authors of the manuscript as indicated below.

NAME OF JOURNAL: Oncogenesis

TITLE OF MANUSCRIPT: Co-activation of Sonic hedgehog and Wnt signaling in murine retinal precursor cells drives ocular lesions with features of intraocular medulloepithelioma

MANUSCRIPT NUMBER: MS#ONCSIS-21-0275-T

CORRESPONDING AUTHORS NAME: Prof. Dr. Julia E. Neumann

PREVIOUS AUTHOR NAMES:

Matthias Dottermusch*, Piotr Sumislawski*, Julia Krevet, Maximilian Middelkamp, Hannah Voß, Harald Bartsch, Karl Sotlar, Andrey Korshunov, Markus Glatzel, Ulrich Schüller, Julia E. Neumann

UPDATED AUTHOR NAMES:

Matthias Dottermusch*, Piotr Sumislawski*, Julia Krevet, Maximilian Middelkamp, Hannah Voß, Bente Siebels, Harald Bartsch, Karl Sotlar, Peter Meyer, Stephan Frank, Andrey Korshunov, Markus Glatzel, Ulrich Schüller, Julia E. Neumann

CHANGE TO AUTHOR LIST:

addition of 3 coauthors: Bente Siebels,Peter Meyer, Stephan Frank

Print Name	Signature	Date
Karl Sotlar	[signature]	13.10.2021

SPRINGER NATURE

In accordance with Springer Nature Authorship Policy we agree to change the authors of the manuscript as indicated below.

NAME OF JOURNAL: Oncogenesis

TITLE OF MANUSCRIPT: Co-activation of Sonic hedgehog and Wnt signaling in murine retinal precursor cells drives ocular lesions with features of intraocular medulloepithelioma

MANUSCRIPT NUMBER: MS#ONCSIS-21-0275-T

CORRESPONDING AUTHORS NAME: Prof. Dr. Julia E. Neumann

PREVIOUS AUTHOR NAMES:

Matthias Dottermusch*, Piotr Sumislawski*, Julia Krevet, Maximilian Middelkamp, Hannah Voß, Harald Bartsch, Karl Sotlar, Andrey Korshunov, Markus Glatzel, Ulrich Schüller, Julia E. Neumann

UPDATED AUTHOR NAMES:

Matthias Dottermusch*, Piotr Sumislawski*, Julia Krevet, Maximilian Middelkamp, Hannah Voß, Bente Siebels, Harald Bartsch, Karl Sotlar, Peter Meyer, Stephan Frank, Andrey Korshunov, Markus Glatzel, Ulrich Schüller, Julia E. Neumann

CHANGE TO AUTHOR LIST:

addition of 3 coauthors: Bente Siebels,Peter Meyer, Stephan Frank

Print Name	Signature	Date
Stephan Frank	[signature] Universitätsspital Basel Pathologie Prof. Dr. med. Stephan Frank Abteilungsleiter Neuro- und Ophthalmopathologie Schönbeinstrasse 40, CH - 4031 Basel Tel. +41 61 328 63 90, Fax +41 61 265 31 94	Oct 13, 2021

SPRINGER NATURE

In accordance with Springer Nature Authorship Policy we agree to change the authors of the manuscript as indicated below.

NAME OF JOURNAL: Oncogenesis

TITLE OF MANUSCRIPT: Co-activation of Sonic hedgehog and Wnt signaling in murine retinal precursor cells drives ocular lesions with features of intraocular medulloepithelioma

MANUSCRIPT NUMBER: MS#ONCSIS-21-0275-T

CORRESPONDING AUTHORS NAME: Prof. Dr. Julia E. Neumann

PREVIOUS AUTHOR NAMES:

Matthias Dottermusch*, Piotr Sumislawski*, Julia Krevet, Maximilian Middelkamp, Hannah Voß, Harald Bartsch, Karl Sotlar, Andrey Korshunov, Markus Glatzel, Ulrich Schüller, Julia E. Neumann

UPDATED AUTHOR NAMES:

Matthias Dottermusch*, Piotr Sumislawski*, Julia Krevet, Maximilian Middelkamp, Hannah Voß, Bente Siebels, Harald Bartsch, Karl Sotlar, Peter Meyer, Stephan Frank, Andrey Korshunov, Markus Glatzel, Ulrich Schüller, Julia E. Neumann

CHANGE TO AUTHOR LIST:

addition of 3 coauthors: Bente Siebels,Peter Meyer, Stephan Frank

Print Name	Signature	Date
Harald Bartsch	[signature]	18.10.21

SPRINGER NATURE

In accordance with Springer Nature Authorship Policy we agree to change the authors of the manuscript as indicated below.

NAME OF JOURNAL: Oncogenesis

TITLE OF MANUSCRIPT: Co-activation of Sonic hedgehog and Wnt signaling in murine retinal precursor cells drives ocular lesions with features of intraocular medulloepithelioma

MANUSCRIPT NUMBER: MS#ONCSIS-21-0275-T

CORRESPONDING AUTHORS NAME: Prof. Dr. Julia E. Neumann

PREVIOUS AUTHOR NAMES:

Matthias Dottermusch*, Piotr Sumislawski*, Julia Krevet, Maximilian Middelkamp, Hannah Voß, Harald Bartsch, Karl Sotlar, Andrey Korshunov, Markus Glatzel, Ulrich Schüller, Julia E. Neumann

UPDATED AUTHOR NAMES:

Matthias Dottermusch*, Piotr Sumislawski*, Julia Krevet, Maximilian Middelkamp, Hannah Voß, Bente Siebels, Harald Bartsch, Karl Sotlar, Peter Meyer, Stephan Frank, Andrey Korshunov, Markus Glatzel, Ulrich Schüller, Julia E. Neumann

CHANGE TO AUTHOR LIST:

addition of 3 coauthors: Bente Siebels,Peter Meyer, Stephan Frank

Print Name	Signature	Date
Andrey Korshunov	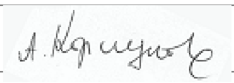	08.10.2021

SPRINGER NATURE

In accordance with Springer Nature Authorship Policy we agree to change the authors of the manuscript as indicated below.

NAME OF JOURNAL: Oncogenesis

TITLE OF MANUSCRIPT: Co-activation of Sonic hedgehog and Wnt signaling in murine retinal precursor cells drives ocular lesions with features of intraocular medulloepithelioma

MANUSCRIPT NUMBER: MS#ONCSIS-21-0275-T

CORRESPONDING AUTHORS NAME: Prof. Dr. Julia E. Neumann

PREVIOUS AUTHOR NAMES:

Matthias Dottermusch*, Piotr Sumislawski*, Julia Krevet, Maximilian Middelkamp, Hannah Voß, Harald Bartsch, Karl Sotlar, Andrey Korshunov, Markus Glatzel, Ulrich Schüller, Julia E. Neumann

UPDATED AUTHOR NAMES:

Matthias Dottermusch*, Piotr Sumislawski*, Julia Krevet, Maximilian Middelkamp, Hannah Voß, Bente Siebels, Harald Bartsch, Karl Sotlar, Peter Meyer, Stephan Frank, Andrey Korshunov, Markus Glatzel, Ulrich Schüller, Julia E. Neumann

CHANGE TO AUTHOR LIST:

addition of 3 coauthors: Bente Siebels,Peter Meyer, Stephan Frank

Print Name	Signature	Date
Hannah Voß	H. Voß	03.10.2021

In accordance with Springer Nature Authorship Policy we agree to change the authors of the manuscript as indicated below.

NAME OF JOURNAL: Oncogenesis

TITLE OF MANUSCRIPT: Co-activation of Sonic hedgehog and Wnt signaling in murine retinal precursor cells drives ocular lesions with features of intraocular medulloepithelioma

MANUSCRIPT NUMBER: MS#ONCSIS-21-0275-T

CORRESPONDING AUTHORS NAME: Prof. Dr. Julia E. Neumann

PREVIOUS AUTHOR NAMES:

Matthias Dottermusch*, Piotr Sumislawski*, Julia Krevet, Maximilian Middelkamp, Hannah Voß, Harald Bartsch, Karl Sotlar, Andrey Korshunov, Markus Glatzel, Ulrich Schüller, Julia E. Neumann

UPDATED AUTHOR NAMES:

Matthias Dottermusch*, Piotr Sumislawski*, Julia Krevet, Maximilian Middelkamp, Hannah Voß, Bente Siebels, Harald Bartsch, Karl Sotlar, Peter Meyer, Stephan Frank, Andrey Korshunov, Markus Glatzel, Ulrich Schüller, Julia E. Neumann

CHANGE TO AUTHOR LIST:

addition of 3 coauthors: Bente Siebels,Peter Meyer, Stephan Frank

Print Name	Signature	Date
Markus Glatzel	M. Gl	04.10.2021

SPRINGER NATURE

In accordance with Springer Nature Authorship Policy we agree to change the authors of the manuscript as indicated below.

NAME OF JOURNAL: Oncogenesis

TITLE OF MANUSCRIPT: Co-activation of Sonic hedgehog and Wnt signaling in murine retinal precursor cells drives ocular lesions with features of intraocular medulloepithelioma

MANUSCRIPT NUMBER: MS#ONCSIS-21-0275-T

CORRESPONDING AUTHORS NAME: Prof. Dr. Julia E. Neumann

PREVIOUS AUTHOR NAMES:

Matthias Dottermusch*, Piotr Sumislawski*, Julia Krevet, Maximilian Middelkamp, Hannah Voß, Harald Bartsch, Karl Sotlar, Andrey Korshunov, Markus Glatzel, Ulrich Schüller, Julia E. Neumann

UPDATED AUTHOR NAMES:

Matthias Dottermusch*, Piotr Sumislawski*, Julia Krevet, Maximilian Middelkamp, Hannah Voß, Bente Siebels, Harald Bartsch, Karl Sotlar, Peter Meyer, Stephan Frank, Andrey Korshunov, Markus Glatzel, Ulrich Schüller, Julia E. Neumann

CHANGE TO AUTHOR LIST:

addition of 3 coauthors: Bente Siebels,Peter Meyer, Stephan Frank

Print Name	Signature	Date
ULRICH SCHÜLLER	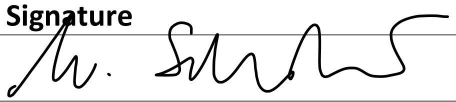	03.10.2021
